# ﻿Taxonomic review of the *Calotesversicolor* complex (Agamidae, Sauria, Squamata) in China, with description of a new species and subspecies

**DOI:** 10.3897/zookeys.1187.110704

**Published:** 2023-12-20

**Authors:** Yong Huang, Hongyu Li, Yilin Wang, Maojin Li, Mian Hou, Bo Cai

**Affiliations:** 1 Guangxi University of Chinese Medicine, Nanning 530200, Guangxi, China Guangxi University of Chinese Medicine Nanning China; 2 Chengdu Institute of Biology, Chinese Academy of Sciences, Chengdu 610041, Sichuan, China Key Laboratory of Protection and Utilization of Traditional Chinese Medicine and Ethnic Medicine Resources, Education Department of Guangxi Zhuang Autonomous Region Nanning China; 3 Hainan Forestry Group Co., Ltd, Haikou 570203, Hainan, China Hainan Forestry Group Co., Ltd Haikou China; 4 College of Continuing (Online) Education, Sichuan Normal University, Chengdu 610068, Sichuan, China Sichuan Normal University Chengdu China; 5 Key Laboratory of Protection and Utilization of Traditional Chinese Medicine and Ethnic Medicine Resources, Education Department of Guangxi Zhuang Autonomous Region, Nanning 530200, Guangxi, China Chengdu Institute of Biology, Chinese Academy of Sciences Chengdu China

**Keywords:** *Calotesirawadi*, *Caloteswangi* sp. nov., garden lizard, southern China, taxonomic review

## Abstract

*Caloteswangi***sp. nov.**, a new species of the agamid genus *Calotes* Cuvier, 1817, from southern China and northern Vietnam, is described. This species can be distinguished from all known congeners by a combination of morphological characteristics and genetic divergence in the mitochondrial tRNA, ND2, and CO1 genes. Molecular phylogenetic analysis revealed that the new species was formed as a monophyletic group and that considerable genetic divergence existed between its congeners (minimum *p*-distance, 4.6%). *Caloteswangi***sp. nov.** is distinguished by a combination of the following characteristics: average SVL < 90 mm for adult males; 10–14 dorsal eyelid scales; scales on side of neck and adjacent shoulder area pointing obliquely upward; keels on neck scales weakly to strongly developed; fold in front of the shoulder absent; pair of dark triangular patches extending from the front of the shoulder to the jaw angles; and orange coloration of the tongue. *Caloteswangi***sp. nov.** is similar to *C.irawadi* but differs in having scales between the nasal shield and the orbit and a fourth toe with a claw that can reach between the eyes and tympanum (even to the snout when hind the limbs are adpressed forward). Phylogenetic analyses revealed two well-supported subspecies, Lineages A and B in *C.wangi***sp. nov.**, with mean uncorrected *p*-distances between them of 2%. We propose that Lineage A, which is mainly from the central and southern Wuzhi Mountains on Hainan Island, is a subspecies, *C.w.hainanensis***ssp. nov.** Lineage B mainly comprises individuals from other sites on the island plus the adjacent mainland, and is described as subspecies, *C.w.wangi***ssp. nov.** A diagnostic key to all *Calotes* species of China is also provided.

## ﻿Introduction

The genus *Calotes* Cuvier, 1817, contains at least 29 species throughout the world, but they are distributed primarily in southern and southeastern Asia (Uetz et al. 2023). Most species of this genus have narrow geographic distributions except *Calotesversicolor* Daudin, 1802, which is mainly found across continental Asia from southeastern Iran in the west to southern China and Indonesia in the east. In China, this species is thought to occur in Yunnan Province, Guangdong Province, Guangxi Zhuang Autonomous Region, Hainan Province, Hunan Province, Fujian Province, Hong Kong, and Macao (Cai et al. 2022a).

*Calotesversicolor* has a complicated taxonomic history. One of the main causes of this complexity is that the original description was based on coloration data and was not detailed enough to distinguish the species (Daudin 1802). Another complication is that two type specimens of *C.versicolor* in the Muséum National d’Histoire Naturelle, Paris (MNHN) have been lost, leaving this taxon without valid types or a type locality (Wermuth 1967; Amarasinghe et al. 2009; Gowande et al. 2016). Kuhl (1820) claimed Pondicherry as the type locality (terra typica) of *C.versicolor*, and this was corroborated by Smith (1935) who stated that it was distributed across the Indian and Indo-Chinese sub-regions including Afghanistan, Ceylon, the Andaman Islands, Pulo Condore (Côn Sơn Island), south China (including Hainan and Hong Kong), and the Malayan peninsula except Sumatra. However, many studies have found that different populations of this species have different morphological characteristics (Smith 1935; Zug et al. 2006; Gowande et al. 2016, 2021), making *C.versicolor* a species complex. It is difficult to study the taxonomy of this species complex in the absence of a valid type specimen and unambiguous type locality.

In 2016, an adult male from Pondicherry was collected and designated as the neotype of *C.versicolor* (NCBS AT102) (Gowande et al. 2016); but it was invalidated by Chaitanya et al. (2017) for several reasons. Gowande et al. (2021) revised *C.versicolor* in the Indian subcontinent, responding to the critique of Chaitanya et al. (2017) and insisting that the neotype of *C.versicolor* and the type locality be restricted to southern and eastern India.

In China, Huang et al. (2013) found that the population of *Calotesversicolor* in south China was different. Liu et al. (2021) reported that the species previously identified as *C.versicolor* from western Yunnan (Dehong Prefecture) was *C.irawadi* Zug, Brown, Schulte & Vindum, 2006. Other populations of *C.versicolor* in China have yet to be clarified.

From 2009 to 2022, we conducted a series of field surveys in south China and collected 323 specimens of this species complex from Fujian Province, Guangdong Province, Guangxi Zhuang Autonomous Region, Hainan Province, Hong Kong, and Yunnan Province in China, and also examined specimens collected from Fujian Province, Guangdong Province, Guangxi Zhuang Autonomous Region, Hainan Province, Hong Kong, and Yunnan Province in China, and from Myanmar and Vietnam. Based on molecular phylogenetic analyses and morphological comparisons, we believe that these specimens include one new undescribed species and two subspecies, which are described herein.

## ﻿Materials and methods

### ﻿Sampling

**Figure 1.**
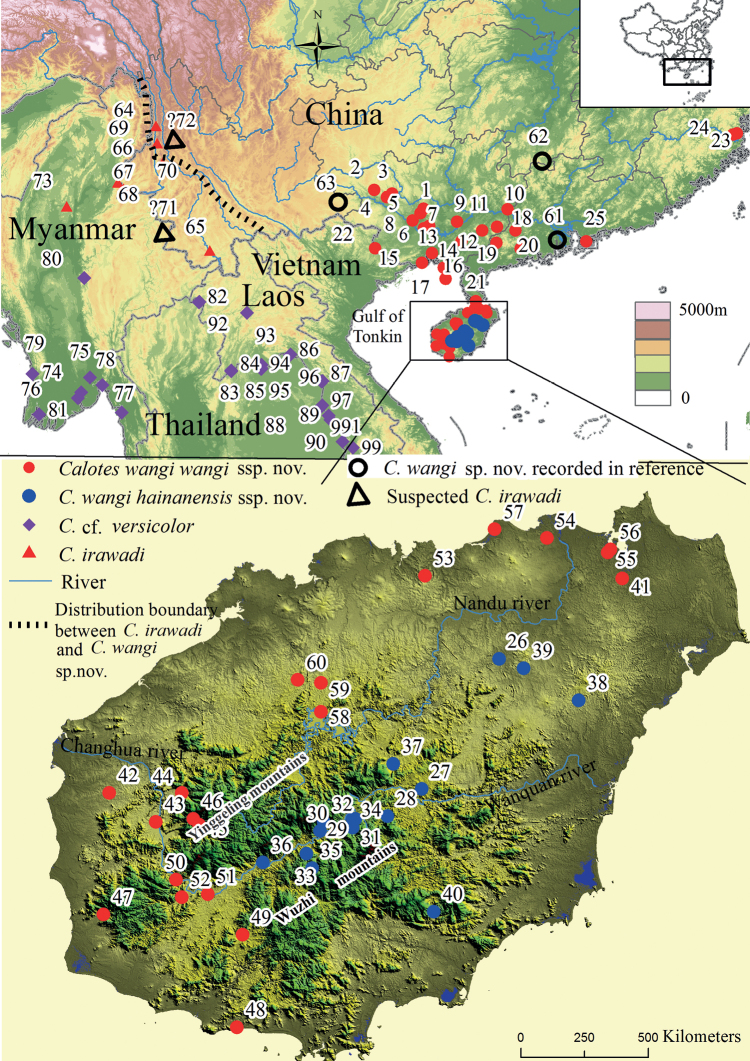
Map [GS(2020)4619] showing representative localities for *Caloteswangi* sp. nov. and morphologically similar species. Color codes: solid red circles *C.w.wangi* ssp. nov., hollow black circles *C.w.wangi*, solid blue circles *C.w.hainanensis* ssp. nov., solid red triangles *C.irawadi*, hollow black triangles suspected *C.irawadi*, solid purple squares C.cf.versicolor. Samples are numbered following Table 1 and Suppl. material 1: table S1.

Fieldwork was carried out around Yunnan Province,Guangdong Province, Fujian Province , and Hainan provinces and Guangxi Zhuang Autonomous Region (Fig. 1, Table 1) from 2009 to 2022 by members of our team, and 323 specimens were collected. Then we took photographs of live animals and measured them. Muscle tissue samples were taken, fixed, and stored at -20 °C in absolute ethanol. The type specimens were fixed in 10% formalin, stored in 75% ethanol, and deposited in the herpetological collections of the Chengdu Institute of Biology, Chinese Academy of Sciences (**CIB**). The specimens were deposited at the **CIB**, Guangxi University of Chinese Medicine (**GXUCM**), Kunming Natural History Museum of Zoology, Kunming Institute of Zoology, Chinese Academy of Sciences (**KIZ**).

**Table 1.** Voucher specimens and GenBank accession numbers of DNA sequences of Caloteswangi sp. nov. used in this study. More species information covered in figure 1 can be found in Suppl. material 1: table S1.

ID	Species	Subspecies	Locality (Abbreviation)	Voucher number	GenBank number	Population number	GPS Coordinates (Latitude, Longitude)	References
1	*Caloteswangi* sp. nov.	*C.w.wangi* ssp. nov.	Mt. Daming, Guangxi, China (DM)	2022091533~2022091541	OR828811~OR828819	9	23.548581, 108.353307	This study
2	*C.wangi* sp. nov.	*C.w.wangi* ssp. nov.	Taohua, Guangxi, China (TH)	201606139~201606142	OR828796~OR828798	3	24.212539, 106.611948	This study
3	*C.wangi* sp. nov.	*C.w.wangi* ssp. nov.	Bama, Guangxi, China (BM)	201606144~201606148	OR828799~OR828803	5	24.09138, 107.250248	This study
4	*C.wangi* sp. nov.	*C.w.wangi* ssp. nov.	Naheng, Guangxi, China (NH)	201606143	OR828804	1	23.953857, 107.065068	This study
5	*C.wangi* sp. nov.	*C.w.wangi* ssp. nov.	Fucheng, Guangxi, China (FC)	201604082~201604087	OR828805~OR828810	6	23.391183, 108.247992	This study
6	*C.wangi* sp. nov.	*C.w.wangi* ssp. nov.	Nanning, Guangxi, China (NN)	HC201002279~HC201002281	KC87576, KC875761, KC875763	3	22.86, 108.37	Huang et al. 2013
7	*C.wangi* sp. nov.	*C.w.wangi* ssp. nov.	Wutang, Guangxi, China (WT)	201511048–201511050, 201511052	OR828820~OR828823	4	22.945312, 108.555563	This study
8	*C.wangi* sp. nov.	*C.w.wangi* ssp. nov.	Dingdang, Guangxi, China (DD)	201604088~201604102	OR828824~OR828838	15	23.13039, 107.976043	This study
9	*C.wangi* sp. nov.	*C.w.wangi* ssp. nov.	Gangbei, Guangxi, China (GB)	201606104~201606108	OR828839~OR828843	5	23.093115, 109.540017	This study
10	*C.wangi* sp. nov.	*C.w.wangi* ssp. nov.	Wuzhou, Guangxi, China (WZ)	201606134~201606137	OR828844~OR828847	4	23.526721, 111.329018	This study
11	*C.wangi* sp. nov.	*C.w.wangi* ssp. nov.	Cenxi, Guangxi, China (CX)	201606130~201606132	OR828848~OR828850	3	22.914898, 110.958258	This study
12	*C.wangi* sp. nov.	*C.w.wangi* ssp. nov.	Rongxi, Guangxi, China (RX)	201606115~201606119	OR828851~OR828855	5	22.784373, 110.43628	This study
13	*C.wangi* sp. nov.	*C.w.wangi* ssp. nov.	Qinnanqu, Guangxi, China (QN)	201510030–201510033, 201510035	OR828856~OR828860	5	21.980953, 108.653817	This study
14	*C.wangi* sp. nov.	*C.w.wangi* ssp. nov.	Wenming, Guangxi, China (WM)	201512053, 201512056	OR828861~OR828862	2	22.4119, 109.6998	This study
15	*C.wangi* sp. nov.	*C.w.wangi* ssp. nov.	Fangchenggang, Guangxi, China (FCG)	201510039–201510044, 201510047	OR828863~OR828869	7	21.635534, 108.301372	This study
16	*C.wangi* sp. nov.	*C.w.wangi* ssp. nov.	Yinhaiqu, Guangxi, China (YH)	201509019~201509020, 201509022~201509025	OR828870~OR828875	6	21.468197, 109.078404	This study
17	*C.wangi* sp. nov.	*C.w.wangi* ssp. nov.	Weizhoudao, Guangxi, China (WZD)	201509003~201509007, 201509009, 201509011, 201509012, 201509014~201509017, 201509027, 201509028	OR822208~OR822221	14	21.066718, 109.139317	This study
18	*C.wangi* sp. nov.	*C.w.wangi* ssp. nov.	Fuchao, Guangdong, China (FCC)	201606125~201606129	OR828876~OR828879	4	22.781087, 111.608735	This study
19	*C.wangi* sp. nov.	*C.w.wangi* ssp. nov.	Xinyi, Guangdong, China (XY)	201606120~201606123	OR828880~OR828883	4	22.339085, 110.937615	This study
20	*C.wangi* sp. nov.	*C.w.wangi* ssp. nov.	Yangchun, Guangdong, China (YC)	201606109~201606114	OR828884~OR828889	6	22.141142, 111.78446	This study
21	*C.wangi* sp. nov.	*C.w.wangi* ssp. nov.	Haian, Guangdong, China (HA)	HC200908192~HC200908199, HCL200908276	KC875759, KC875749~KC875756	9	20.28, 110.21	Huang et al. 2013
22	*C.wangi* sp. nov.	*C.w.wangi* ssp. nov.	Lang Son, Vietnam (LS)	HC201006282~HC201006288	KC875765~KC87577	7	22.15, 106.65	Huang et al 2013
23	*C.wangi* sp. nov.	*C.w.wangi* ssp. nov.	Lianjiang, Fujian, China (LJ)	/	/		26.2189, 119.4314	Hu et al. 2022
24	*C.wangi* sp. nov.	*C.w.wangi* ssp. nov.	Jin’an, Fujian, China (JA)	2022091526–2022091527	OR878647~OR878648	2	26.175443, 119.296059	This study
25	*C.wangi* sp. nov.	*C.w.wangi* ssp. nov.	Hongkong, China (HK)	HC201006295	KC875772	1	22.4, 114.11	Huang et al. 2013
26	*C.wangi* sp. nov.	*C.w.hainanensis* ssp. nov.	Tunchang, Hainan, China (TC)	HCL200907005~HCL200907007	KC875611~KC875613	3	19.58298, 110.17577	Huang et al. 2013
27	*C.wangi* sp. nov.	*C.w.hainanensis* ssp. nov.	Wanling, Hainan, China (WL)	HCL200907047~HCL200907052	KC875614~KC875619	6	19.13316, 109.90797	Huang et al. 2013
28	*C.wangi* sp. nov.	*C.w.hainanensis* ssp. nov.	Jiachai, Hainan, China (JC)	HCL200907053~HCL200907054	KC875620~KC875621	2	19.04, 109.79	Huang et al. 2013
29	*C.wangi* sp. nov.	*C.w.hainanensis* ssp. nov.	Hongmao, Hainan, China (HM)	HCL200907055, HCL200908071	KC875622, KC875638	2	19.03, 109.68	Huang et al. 2013
30	*C.wangi* sp. nov.	*C.w.hainanensis* ssp. nov.	Chonggongbao, Hainan, China (CG)	HCL200908056~HCL200908061	KC875623~KC875628	6	18.9886, 109.55716	Huang et al. 2013
31	*C.wangi* sp. nov.	*C.w.hainanensis* ssp. nov.	Fanxiang, Hainan, China (FX)	HCL200908062~HCL200908068	KC875629~KC875635	7	19.03, 109.67	Huang et al. 2013
32	*C.wangi* sp. nov.	*C.w.hainanensis* ssp. nov.	Zayun, Hainan, China (SY)	HCL200908069	KC875636	1	19.02, 109.57	Huang et al. 2013
33	*C.wangi* sp. nov.	*C.w.hainanensis* ssp. nov.	Maoyang, Hainan, China (MY)	HCL200908070	KC875637	1	18.91, 109.51	Huang et al. 2013
34	*C.wangi* sp. nov.	*C.w.hainanensis* ssp. nov.	Hela, Hainan, China (HL)	HCL200908072~HCL200908075	KC875639~KC875642	4	19, 109.67	Huang et al. 2013
35	*C.wangi* sp. nov.	*C.w.hainanensis* ssp. nov.	Hongshan, Hainan, China (HS)	HCL200908076~HCL200908082	KC875643~KC875649	7	18.86, 109.53	Huang et al. 2013
36	*C.wangi* sp. nov.	*C.w.hainanensis* ssp. nov.	Fanyang, Hainan, China (FY)	HCL200908083~HCL200908089	KC875650~KC875656	7	18.88, 109.36	Huang et al. 2013
37	*C.wangi* sp. nov.	*C.w.hainanensis* ssp. nov.	Limushan, Hainan, China (LMS)	HCL200908168~HCL200908172	KC875731~KC875735	5	19.22, 109.81	Huang et al. 2013
38	*C.wangi* sp. nov.	*C.w.hainanensis* ssp. nov.	Huangzhu, Hainan, China (HZ)	HCL200908181~HCL200908184	KC875741~KC875744	4	19.44, 110.45	Huang et al. 2013
39	*C.wangi* sp. nov.	*C.w.hainanensis* ssp. nov.	Fuwen, Hainan, China (FW)	CIB091425~CIB091426	KC875778,KC875777	2	19.55, 110.26	Huang et al. 2013
40	*C.wangi* sp. nov.	*C.w.hainanensis* ssp. nov.	Lingshui, Hainan, China (LS)	CIB91435~CIB91452	KC875787~KC875804	18	18.71, 109.95	Huang et al. 2013
41	*C.wangi* sp. nov.	*C.w.wangi* ssp. nov.	Wenchang, Hainan, China (WC)	HC200907002	KC875610	1	19.86, 110.6	Huang et al. 2013
42	*C.wangi* sp. nov.	*C.w.wangi* ssp. nov.	Datian, Hainan, China (DT)	HCL200908091~HCL200908092, HCL200908094~HCL200908098	KC875657~KC875663	7	19.12, 108.83	Huang et al. 2013
43	*C.wangi* sp. nov.	*C.w.wangi* ssp. nov.	Donghe, Hainan, China (DH)	HCL200908099~HCL200908104	KC875664~KC875669	6	19.02, 108.99	Huang et al. 2013
44	*C.wangi* sp. nov.	*C.w.wangi* ssp. nov.	Wanting, Hainan, China (WT)	HCL200908105~HCL200908109	KC875670~KC875674	5	19.12, 109.08	Huang et al. 2013
45	*C.wangi* sp. nov.	*C.w.wangi* ssp. nov.	Sanpai, Hainan, China (SP)	HCL200908110~HCL200908121, HCL200908200, HC200908278	KC875675~KC875760	13	19.01, 109.14	Huang et al. 2013
46	*C.wangi* sp. nov.	*C.w.wangi* ssp. nov.	Bawangling, Hainan, China (BWL)	HCL200908122~HCL200908127	KC875687~KC87569	6	19.03, 109.12	Huang et al. 2013
47	*C.wangi* sp. nov.	*C.w.wangi* ssp. nov.	Jianfeng, Hainan, China (JF)	HCL200908128~HCL200908134	KC875693~KC875699	7	18.7, 108.81	Huang et al. 2013
48	*C.wangi* sp. nov.	*C.w.wangi* ssp. nov.	Tianya, Hainan, China (TY)	HCL200908136~HCL200908139, HCL200908141~HCL200908146, HCL200908275	KC875758, KC875708~KC875709, KC8757095~KC8757097, KC8757092, KC875700~KC875704	11	18.31, 109.27	Huang et al. 2013
49	*C.wangi* sp. nov.	*C.w.wangi* ssp. nov.	Zhizhong, Hainan, China (ZZ)	HCL200908147~HCL200908153	KC875710~KC875716	7	18.63, 109.29	Huang et al. 2013
50	*C.wangi* sp. nov.	*C.w.wangi* ssp. nov.	Jiangbian, Hainan, China (JB)	HCL200908154~HCL200908155	KC875717~KC875718	2	18.82, 109.06	Huang et al. 2013
51	*C.wangi* sp. nov.	*C.w.wangi* ssp. nov.	Yongming, Hainan, China (YM)	HCL200908156~HCL200908159	KC875719~KC875722	4	18.77, 109.17	Huang et al. 2013
52	*C.wangi* sp. nov.	*C.w.wangi* ssp. nov.	Zhiwei, Hainan, China (ZW)	HCL200908160~HCL200908167	KC875723~ KC875730	8	18.76, 109.08	Huang et al. 2013
53	*C.wangi* sp. nov.	*C.w.wangi* ssp. nov.	Fushan, Hainan, China (FS)	HCL200908173~HCL200908178	KC875736~KC875740	5	19.87, 109.92	Huang et al. 2013
54	*C.wangi* sp. nov.	*C.w.wangi* ssp. nov.	Haikou, Hainan, China (HK)	HC200907001, HCL200908185~HCL200908191	KC875745~KC875748, KC875609	5	20, 110.34	Huang et al. 2013
55	*C.wangi* sp. nov.	*C.w.wangi* ssp. nov.	Yanfeng1, Hainan, China (YF1)	CIB091420	KC875773	1	19.95, 110.55	Huang et al. 2013
56	*C.wangi* sp. nov.	*C.w.wangi* ssp. nov.	Yanfeng2, Hainan, China (YF2)	CIB091421, CIB091423	KC875774~ KC875775	2	19.96, 110.56	Huang et al. 2013
57	*C.wangi* sp. nov.	*C.w.wangi* ssp. nov.	Changliu, Hainan, China (CL)	CIB091424	KC875776	1	20.03, 110.16	Huang et al. 2013
58	*C.wangi* sp. nov.	*C.w.wangi* ssp. nov.	Nanfeng, Hainan, China (NF)	CIB091427~CIB091433	KC875779~KC875785	7	19.4, 109.56	Huang et al. 2013
59	*C.wangi* sp. nov.	*C.w.wangi* ssp. nov.	Nada1, Hainan, China (ND1)	CIB091434	KC875786	1	19.5, 109.56	Huang et al. 2013
60	*C.wangi* sp. nov.	*C.w.wangi* ssp. nov.	Nada2, Hainan, China (ND2)	CIB091453~CIB091468	KC875805~KC875820	16	19.51, 109.48	Huang et al. 2013
61	*C.wangi* sp. nov.	*C.w.wangi* ssp. nov.	Macao, China (MC)	/	/	/	22.158855, 113.577999	This study
62	*C.wangi* sp. nov.	*C.w.wangi* ssp. nov.	Yizhang, Hunan, China (YZ)	/	/	/	24.942264, 112.93057	Deng and Ye 1996, 1997
63	*C.wangi* sp. nov.	*C.w.wangi* ssp. nov.	Funing, Yunnan, China (FN)	/	/	/	23.484035, 105.793296	Yang and Rao 2008
64	*C.irawadi*		Mt. Gaoligong, Yunnan, China (GLG)	HC201006290~HC201006291	/	2	26.42, 98.9	Huang et al. 2013
65	*C.irawadi*		Xishuangbanna, Yunnan, China (XSBN)	HC201006292	/	1	22.01, 100.8	Huang et al. 2013

### ﻿Morphological characteristics

Measurements were taken as suggested by Zhao et al. (1999) and Zug et al. (2006): head length (**HeadL**) as distance from the posterior edge of the jaw to the tip of the snout; head height (**HeadH**) measured as the dorsoventral distance from the top of the head to the underside of the jaw at the transverse plane intersecting the angle of the jaws; head width (**HeadW**) measured between the widest points of the temporal or jaw muscles without compression of soft tissue; eye-to-ear length (**EyeEar**), the distance from the anterior edge of the tympanum to the posterior part of the orbit (not the pupil opening); eye diameter (**EyeDiam**), measured between the anterior and posterior edges of the orbital bone; naris-to-eye length (**NarEye**), the distance from the anterior edge of the orbit to the posterior edge of the naris; the snout-eye length (**SnEye**), measured between the tip of the snout and the anterior edge of the orbital bone; interorbital width (**Interorb**), the transverse distance between the anterodorsal corners of the left and right orbits; jaw width (**JawW**), the distance from the left to right outer edges of the jaw angles (excluding broadening of the jaw musculature); snout-to-forelimb length (**SnForeL**), the distance from the anterior part of the forelimb, or shoulder, to the tip of the snout; snout-to-vent length (**SVL**), from the tip of the snout to the anterior edge of the cloaca; trunk length (**TrunkL**), the distance between the posterior edge of the forelimb insertion (axilla) to the anterior edge of the hind limb insertion (inguen); tail length (**Tail**), measured from the anterior edge of the cloaca to the tip of tail; forelimb length (**FLL**), the distance between the point of insertion (axilla) to the tip of finger IV (excluding claw), measured with the limb straight; hind limb length (**HLL**), the distance between the point of insertion at the groin to the tip of toe IV (excluding claw), measured with the limb straight; toe IV length (**4ToeLng**), distance from the tip of toe IV to the base between toe III and IV (excluding claw); finger IV length (**4FingLng**), distance from the tip of finger IV to the base between toe III and IV (excluding claw); upper arm length (**UparmL**), distance from the anterior insertion of the forelimb, or shoulder, to the elbow; lower arm length (**LoArmL**), distance from the elbow to the distal end of the wrist, or just before the underside of the fore-foot; upper leg length (**UpLegL**), distance from the anterior edge of the hind limb insertion to the knee; crus length (**CrusL**), length of tibia from knee to heel; and, snout width (**SnW**), transverse distance between left and right nares. All measurements were recorded to the nearest 1 mm using a steel ruler for SVL and tail, and to the nearest 0.1 mm using a digital caliper for shorter measurements.

Definitions of morphological characters and the counting methods also mainly followed Zug et al. (2006), Gowande et al.(2021), and Cai et al. (2022b): supralabial scale count (**Suplab**), number of enlarged, modified labial scales from the rostral area to the corner of the mouth; infralabial scale count (**Inflab**), number of enlarged, modified labial scales from the mental area to the corner of the mouth; nasal-supralabials scale rows (**NSL**), number of horizontal rows of small scales between the first supralabial and the nasal; suborbital scale rows (**SoR**), number of longitudinal rows of scales between supralabials and inferior-most edge of orbit circle, excluding fine ciliary scales in the orbit; ventral scale count (**VN**), number of ventral body scales counted in a straight line along the medial axis between the transverse gular fold and the anterior edge of cloaca; finger IV subdigital lamellae count (**4FingLm**), number of subdigital lamellar scales from the base between fingers III and IV to the tip of finger IV, excluding the claw; toe IV subdigital lamellae count (**4ToeLm**), number of subdigital lamellar scales from the base between toes III and IV to the tip of toe IV, excluding the claw; post-tympanic scale count (**PtY**), number of enlarged, distinctively-keeled, raised conical, or sub-pyramidal scales posterior to the tympana and superior to the rictus; post-occipital scale count (**PoS**), number of enlarged, distinctively-keeled, raised conical, or sub-pyramidal scales on the occipital region of the head; dorsal scales or spines (**Dorsal**), number of mid-dorsal scales or spines, beginning with the first enlarged spine-like scale on the nape to above the vent; mid-body scale rows (**Mid-bodyS**), number of scale rows around trunk at mid-body; dorsal eyelid scales (Eyelid), number of scales along dorsal edge of eyelid; state of scales on side of neck and adjacent shoulder area (**SSneck**), horizontal or pointing obliquely upward; state of keels on scales of the neck and adjacent shoulder area (**Ksneck**), modestly to strongly developed or weakly to strongly developed; nuchal crest state (**NuchalCrest**), nuchal crest scales slightly or significantly larger than dorsal crest scales; trunk scales state (**TrunkSc**), smaller or larger than or equal in size to ventral scales; dorsal crest scales state (**DorsalCrests**), long or short, positioned extending backwards; state of nuchal crest scales (**NuchalCrests**), long or short, compared with the size of the dorsal crest scales; state of supratympanic spines (**SpinesS**), length in proportion to the tympanic chamber diameter; scales between the nasal shield and the orbit (**NarEyeS**), scales of the loreal region in a single row between the nasal shield and the orbit; hind limbs reach the orbit or tympanic membrane when adpressed forward (**HAF**); parallel rows of compressed scales above tympanum (**ParallelR**), present or absent; postorbital spine (**PostorbitalS**), present or absent; state of the keels on scales of the lateral trunk (**Kstrunk**), pointing obliquely upward or downward; crescent-shaped patch of granular scales in front of forelimb insertion (**GranularS**), present or absent; gular scale count (**GU**), number of gular scales counted in a straight line along medial axis between and excluding mental and transverse gular fold; gular pouch (**GP**), present or absent; fold in front of shoulder (FS), present or absent; gular fold (**GF**), present or absent.

Coloration descriptions used terminology and codes from RGB (red, green, blue) color scale (Ibraheem et al. 2012; Cai et al. 2022b). Data on coloration and ornamentation were also collected from live specimens, and included the following (Gowande et al. 2021; Cai e al. 2022b): radial stripes below eyes (**RSBE**), present or absent; inner-lip coloration (**ILC**); coloration of the oral cavity (**CO**), defined as the background color of the anterior roof and sides of the mouth, excluding the posterior palate and deep throat; coloration of the tongue (**CTG**), defined as the color of the tongue; dark bands on trunk (**TrnkBand**), number of dark bands (bars) on dorsum of trunk between axilla and inguen, excluding bands over shoulder or pelvis; fore- and hindlimb cross-bands (**LimbBand**); tail cross-bands (**TailBand**); ventral trunk striping (**TrunkSt**), striping ventrally on trunk, none (0), irregular or broken striping (1), or continuous striping (2), excluding midline; dorsal bars mid-dorsal state (**DorsalBar**) broad or narrow, offset on opposite sides of dorsal crest or congruent on opposite sides of dorsal crest; paired nuchal spots (**NucSpot**), shape of paired nuchal spots in front of forelimbs; adult male coloration during breeding season (**Malecolor**).

Since the new species, *Caloteswangi* sp. nov., is geographically and phylogenetically close to *C.irawadi*, enhanced morphometric data of the two species was gathered for statistical analyses. We ln-transformed all trait measurements to normalize and then removed allometric effects of body size for each trait measurement/SVL. Principal component analysis (PCA) was used to distinguish the new species from *C.irawadi* and the dimensionality of morphological characteristics measurements was reduced using SPSS22. Due to sexual dimorphism in *C.versicolor* (Wei et al., 2018), we carried out PCA in both males and females.

### ﻿DNA extraction and sequencing

DNA was extracted from muscle tissues using a kit (DP304, Tiangen Biotech Co., Ltd). A mitochondrial DNA fragment spanning the tRNA_Trp_, ND2 (L3705:5’-ATTAGGGTCTGCTACACAAGC AGTTGG-3’, H5162:5’-GGTTGARAGTARTCATCGAGTTAAGAACGAC-3’), and COI (L5037:5’-GAGTAGACCCAGGAACCRAAGTTC-3’, H6448:5’-GTATACCGGCTAATCCAAGCATGT G-3’) was amplified using the primer pairs of Huang et al. (2013).

The polymerase chain reaction (PCR) was carried out in a 25 μL reaction volume containing 1 μL of template DNA (1 ng/μL), 1 μL of each primer (1 μmol each), 2.5 μL of 10×Takara Ex Taq buffer (Mg^2+^Plus), 2 μL dNTPs (2.5 μmol/L each), 0.2 μL of Takara Ex Taq DNA polymerase (5 U/μL), with the volume made up using sterile distilled water. The PCR conditions were initial denaturation step at 95 °C for 4 min, 35 cycles of denaturation at 94 °C for 35 s, annealing at 65 °C for 45 s, extension at 72 °C for 1 min, and final extension at 72 °C for 10 min. PCR products were sequenced using the amplification primers. The sequences were deposited in GenBank (OR822208–OR822221, OR828796–OR828889,OR878647–OR878648). We also obtained sequences of *C.emma* Gray, 1845, *C.liolepis* Boulenger, 1885, and *C.irawadi* from GenBank. We used *Agamaagama* (Linnaeus, 1758) as outgroup. Detailed information on these materials is shown in Table 1, Suppl. material 1: table S1, and Fig. 1.

### ﻿Phylogenetic analyses

The new sequences and all homologous DNA sequences of *Calotes* available in GenBank (Table 1) were aligned by BioEdit 5.0.9 (Hall 1999). We constructed phylogenetic trees using neighbor-joining (NJ), maximum likelihood (ML) and Bayesian inference (BI) methods, respectively. The NJ tree was generated by Mega X (Kumar et al. 2018) with the bootstrap consensus tree inferred from 1000 replicates. Maximum likelihood trees were created using the IQ-TREE web server with 1000 bootstrap alignments (Nguyen et al. 2015; Trifinopoulos et al. 2016). The substitution model was selected using the model selection tool of IQ-TREE. BI was performed using MrBayes 3.2 (Ronquist et al. 2012). We partitioned the data by codon (tRNA, ND2, and COI) and selected the best-fit model of evolution for each partition using the jModelTest (Posada 2008) based on the BI criterion. The best-fitting nucleotide substitution models were HKY+G, TIM2+I+G and TPM3uf+G for tRNA, ND2 and COI, respectively. The random tree used as the starting tree included two independent runs with four Markov chain Monte Carlo simulations (three hot chains and one cold chain), which were repeated for ten million iterations and sampled every 1000 iterations. The initial 25% of samples were discarded as burn-in to build a consistent tree. An uncorrected *p*-distance was determined in Mega X using the default settings (Kumar et al. 2018). We also constructed phylogenetic trees between new sequences in this study and the sequences of neotypes of *C.versicolor* (Gowande et al. 2021) and other *Calotes* species by the NJ, ML, and BI methods using only the COI gene.

## ﻿Results

### ﻿Phylogenetic results

**Figure 2.**
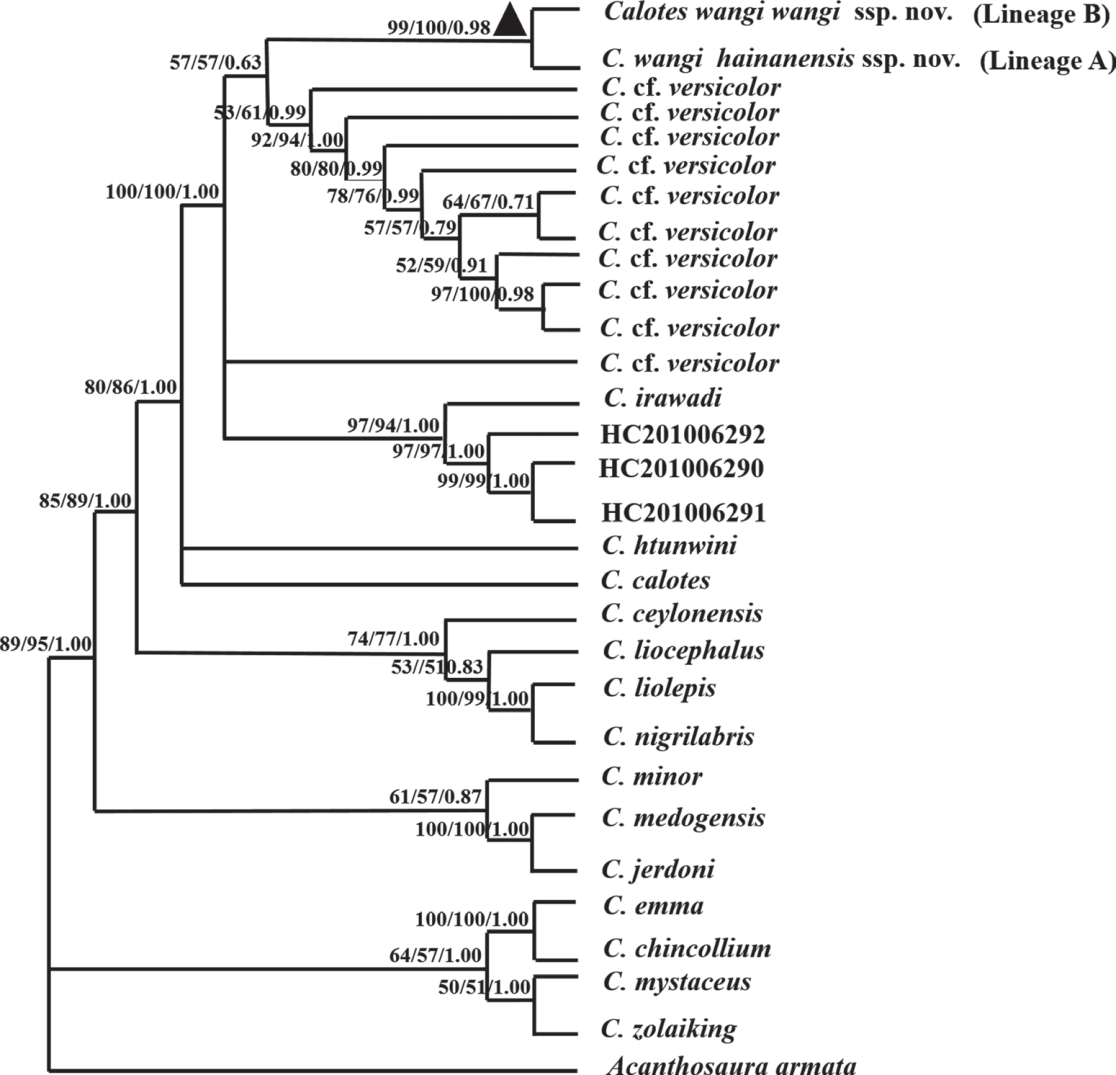
The phylogeny tree of *Caloteswangi* sp. nov. using tRNA_Trp_, ND2, and COI based on neighbor-joining (NJ), maximum likelihood (ML) and Bayesian inference (BI) methods, respectively. The values displayed above the nodes are the NJ, ML, and BI support rates, respectively. Calotescf.versicolor is from Zug et al. (2006). More structural information of phylogenetic trees can be found in Suppl. material 1: fig. S1.

A total of 2663 bp of mitochondrial DNA, spanning tRNA_Trp_, ND2, and COI was studied. NJ, ML and BI analyses showed essentially similar topologies (Fig. 2). The tree result indicated that populations, which were collected from Fujian Province, Guangdong Province, Hainan Province and Guangxi Zhuang Autonomous Region, China and Lang Son, Vietnam, originally assigned to a potential new species formed a monophyletic group with well-supported values (NJ, ML bootstrap values > 90, BI posterior probabilities > 0.9) and a sister taxon with Calotescf.versicolor as mentioned in Zug et al. (2006). The tree result was also consistent with the phylogenic relationship from previous studies with two well separated operational taxonomic units (OTU) ; Lineage A and Lineage B) as reported in Huang et al. (2013). Lineage A is represented by specimens from the central and southern Wuzhi Mountains on Hainan Island, while the other OTU (Lineage B) contains all samples from other sites on Hainan Island and the adjacent Chinese mainland, which form two clearly distinct monophyletic lineages and represent subspecies of this potential species (Fig. 2).

Mean uncorrected *p*-distances between and within species of *Calotes* included in this study are given in Table 2. The *p*-distances between the new species *C.wangi* sp. nov. and C.cf.versicolor, and 14 of the 22 known *Calotes* species, ranged from 4.6 to 26.8%. The intraspecies genetic distance of *C.wangi* sp. nov. was 1.1%. The *p*-distance between Lineage A and Lineage B was 2%. The intraspecies genetic distance of Lineage A and Lineage B was 0.5% and 0.3%, respectively.

**Table 2.** Uncorrected *p*-distances of mitochondrial DNA (tRNA_Trp_, ND2, and COI, 2663 bp) among these new lineages (new species) and other species in the genus *Calotes*.

	*C.emma*	*C.irawadi*	*C.liolepis*	*C.minor*	*C.nigilabris*	*C.mystaceus*	*C.cmedogensis*	*C.liocephalus*	*C.jerdoni*	*C.htunwini*	*C.chincollium*	*C.ceylonensis*	*C.calotes*	*C.zolaiking*	C.cf.versicolor	*C.wangi* sp. nov.
*Calotesemma*	*															
*C.irawadi*	0.231	*														
*C.liolepis*	0.246	0.196	*													
*C.minor*	0.223	0.186	0.204	*												
*C.nigilabris*	0.224	0.170	0.118	0.193	*											
*C.mystaceus*	0.223	0.246	0.226	0.220	0.223	*										
*C.cmedogensis*	0.235	0.197	0.212	0.185	0.218	0.247	*									
*C.liocephalus*	0.227	0.185	0.181	0.195	0.163	0.226	0.204	*								
*C.jerdoni*	0.238	0.194	0.222	0.178	0.218	0.251	0.135	0.196	*							
*C.htunwini*	0.231	0.164	0.209	0.209	0.179	0.232	0.213	0.188	0.220	*						
*C.chincollium*	0.022	0.228	0.237	0.220	0.222	0.210	0.236	0.221	0.236	0.227	*					
*C.ceylonensis*	0.231	0.181	0.190	0.219	0.162	0.244	0.215	0.177	0.207	0.197	0.232	*				
*C.calotes*	0.224	0.147	0.169	0.183	0.150	0.223	0.195	0.161	0.184	0.148	0.222	0.174	*			
*C.zolaiking*	0.255	0.261	0.252	0.245	0.259	0.241	0.250	0.249	0.234	0.268	0.250	0.265	0.234	*		
C.cf.versicolor	0.226	0.057	0.193	0.186	0.179	0.242	0.195	0.187	0.190	0.163	0.222	0.184	0.137	0.254	*	
*C.wangi* sp. nov.	0.228	0.056	0.192	0.185	0.179	0.245	0.203	0.184	0.195	0.164	0.226	0.181	0.142	0.255	0.046	*

**Figure 3.**
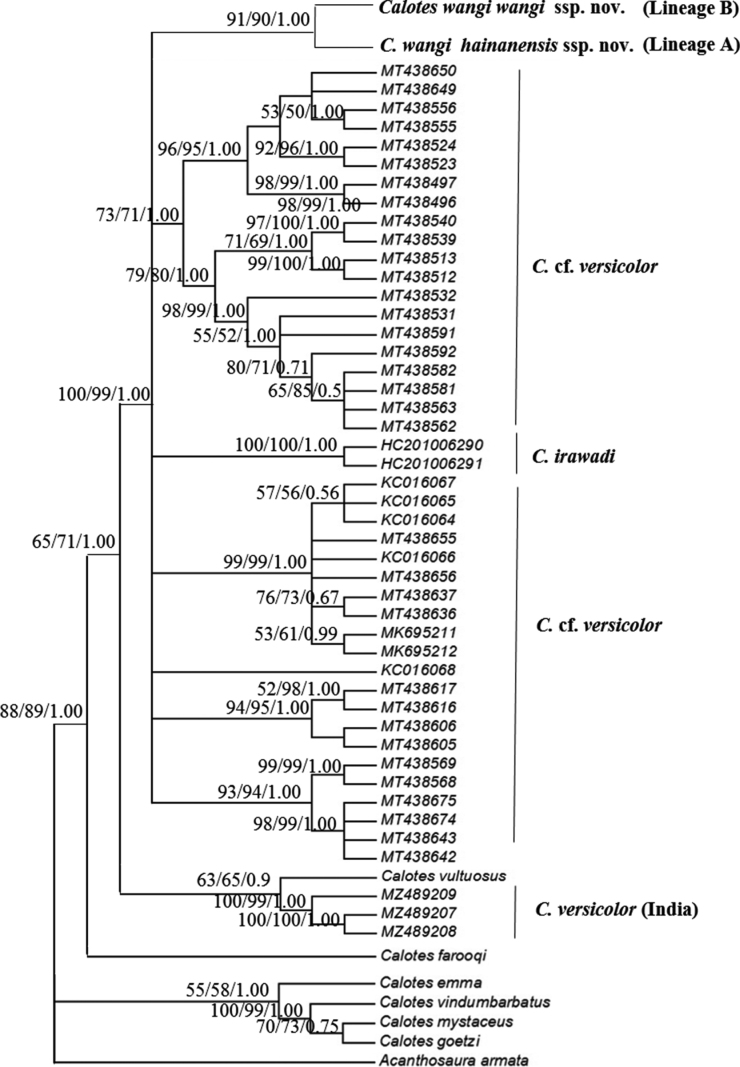
The phylogeny tree of *Caloteswangi* sp. nov. using only COI based on neighbor-joining (NJ), maximum likelihood (ML), and Bayesian inference (BI) methods, respectively. Numbers at the terminals of the branches correspond to the voucher numbers in Table 1. The values displayed above the nodes are the NJ, ML, and BI support rates, respectively.

Based on the COI gene (1257 bp) alone, we combined our data and that of Hartmann et al. (2013), Gowande et al. (2021) and Tantrawatpan et al. (2021) to construct a phylogenic tree by the NJ, ML, and BI methods, respectively. The trees also revealed that our samples of *C.wangi* sp. nov. formed a monophyletic clade with the existence of two deeply divergent OTUs (Lineage A and Lineage B), which were strongly supported (NJ, ML bootstrap values > 90, BI posterior probabilities > 0.9; Fig. 3). The *p*-distances between *C.wangi* sp. nov. and C.cf.versicolor, and the other *Calotes* species ranged from 4.4 to 21.5%. The intraspecies genetic distance of *C.wangi* sp. nov. was 1.3%. The *p*-distance between Lineage A and Lineage B was 2% (Table 3), and the intraspecies genetic distance of Lineage A and Lineage B was 0.5% and 0.3%, respectively.

**Table 3.** Uncorrected *p*-distances of mitochondrial DNA (COI,1257 bp) among these new lineages (new species) and other species in the genus *Calotes*.

	*C.emma*	*C.mystaceus*	*C.farooqi*	*C.vindumbarbatus*	*C.vultuosus*	*C.goetzi*	*C.versicolor*	C.cf.versicolor
*Calotesemma*	*							
*C.mystaceus*	0.154	*						
*C.farooqi*	0.200	0.179	*					
*C.vindumbarbatus*	0.165	0.054	0.188	*				
*C.vultuosus*	0.215	0.192	0.169	0.188	*			
*C.goetzi*	0.167	0.048	0.179	0.077	0.196	*		
*C.versicolor*	0.190	0.188	0.171	0.188	0.134	0.189	*	
C.cf.versicolor	0.200	0.181	0.175	0.184	0.152	0.182	0.139	*
*C.wangi* sp. nov.	0.199	0.177	0.174	0.177	0.151	0.182	0.144	0.044

### ﻿Morphological results

Based on the morphological data and compared with known species or populations in the *Calotesversicolor* complex, *C.wangi* sp. nov. from southern China (except western Yunnan) distinctly differed from the neotype of *C.versicolor* (India) and other *Calotes* species, showing some unique characteristics. The average adult male had SVL < 90 mm, a smaller HeadH/SVL, a larger HindLimbL/SVL, 4ToeLng/SVL and 4FingLng/SVL, NarEyeS < 6; scales on side of trunk, neck and adjacent shoulder area pointing obliquely upward; paired nuchal spots present and extending below the last infralabial scales; the fourth toe with claw can stretch between the eyes and tympanum, and even to the snout when the hind limbs are adpressed forward. Combined with phylogenetic and morphological differences, we conclude that the specimens from southern China (except for western Yunnan) represent a distinct species and subspecies that are described as follows.

**Figure 4.**
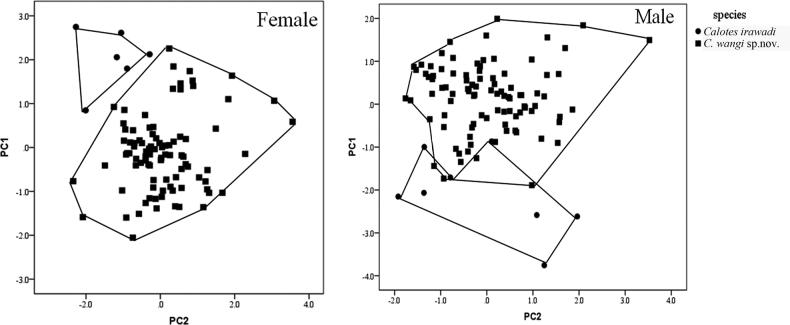
Scatter plots of PC1 and PC2 of principal component analysis (PCA) based on morphometric measurements, distinguishing *Caloteswangi* sp. nov. from *C.irawadi*.

In the PCA results (Fig. 4), the extracted components PC1, PC2, and PC3 accounted for 45.9%, 27.8%, 15.7% for males, and 46.5%, 30.4%, 16.5% for males, respectively. The scatter plots of PC1 and PC2 showed that samples of the new species *C.wangi* sp. nov. and *C.irawadi* were clustered separately and had almost no overlap with each other.

## ﻿Systematics

### ﻿ Calotes wangi sp. nov.

Taxon classificationAnimaliaSquamataAgamidae

CE5F2D45-B1E3-525F-BE31-1C7E06221AF7

https://zoobank.org/FE6F8314-08C6-47F5-AA6D-E97E45D0E765

**Figure 5.**
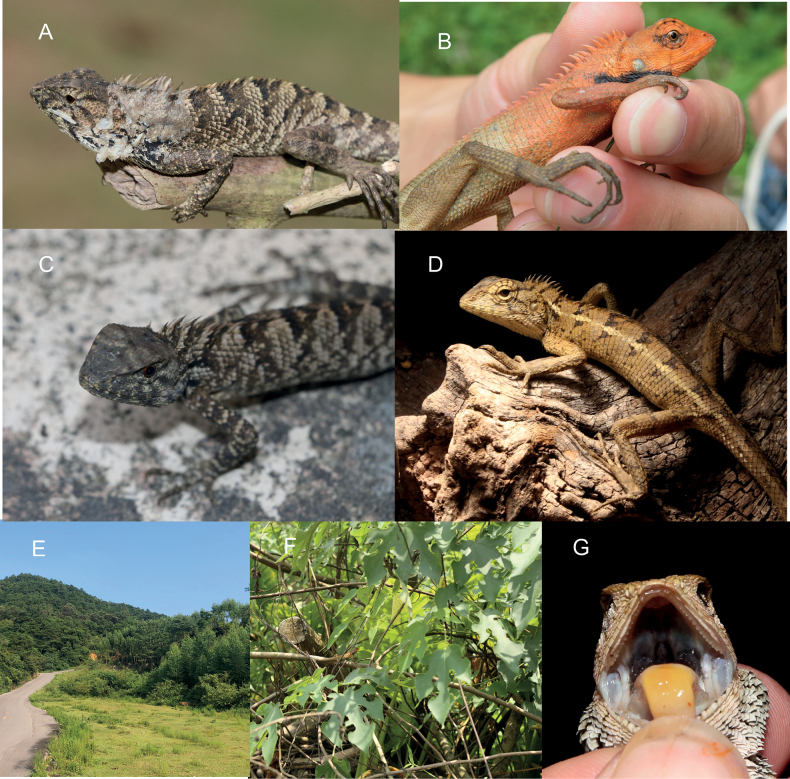
Photographs of live specimens and their habitats **A** holotype of *Caloteswangi*GXUCM-H202291534 **B** holotype of *C.w.hainanensis* CIB095629 **C** allotype of *C.wangi*GXUCM-H202291533 **D** allotype of *C.w.hainanensis* CIB095630 **E, F** habitats **G** oral cavity view.

**Figure 6.**
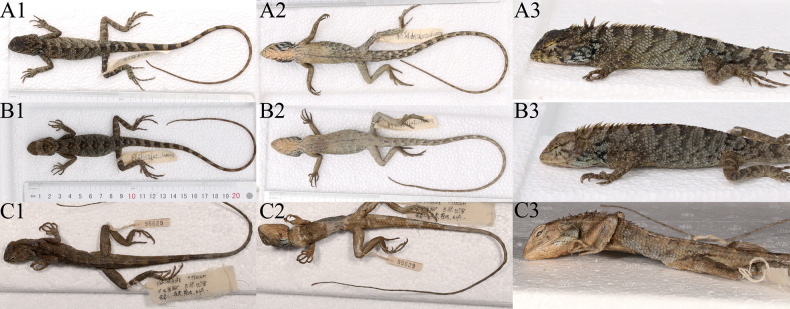
**A** holotype of *Caloteswangi*GXUCM-H202291534, A1, A2, A3 **B** allotype of *C.wangi*GXUCM-H202291533, B1, B2, B3 **C** holotype of *C.w.hainanensis* CIB095629, C1, C2, C3. 1, dorsal view; 2, ventral view, 3; Dorsolateral view.

Figs 5 , 6 , Table 4

Calotes versicolor : Smith 1935: 189–193 in S. China and Hainan; Zhao and Adler 1993: 189 excluding W. Yunnan; Deng and Ye 1996, 1997; Zhao et al. 1999: 97–110 excluding W. Yunnan; Liu 2000; Cai et al. 2015 excluding W. Yunnan; Ge et al. 2018; Wang et al. 2021 excluding W. Yunnan; Cai et al. 2022a excluding W. Yunnan; Hu et al. 2022.

#### Type material.

***Holotype*.** Adult male, CIB119358 (filed number GXUCM-H202291534). Collected from Daming Montains, Wuming District, Nanning City, Guangxi Zhuang Autonomous Region, China (23.52654°N, 108.342559°E, 326 m a. s. l.) by Yong Huang in September 2022.

***Allotype*.** Adult female, CIB119359 (filed number GXUCM-H202291533), with the same locality and collector information as the holotype.

***Paratypes*.** Adult males (GXUCM-H201604082-4, H201604086-87, GXUCM-H2022091535-36) and adult females (GXUCM-H201604085, H2022091538), with the same locality and collector information as the holotype.

#### Other examined specimens.

**China**, **1.** Guangdong Province, Xuwen (1♂), Yangchun (3♂, 2♀), Luoding (2♂, 3♀), Xinyi (3♂, 2♀). **2.** Guangxi Zhuang Autonomous Region, Bama (3♂, 2♀), Yinhai (1♂, 2♀), Weizhou island (13♂, 6♀), Cenxi (3♂, 1♀), Gangkou (1♂, 2♀), Shiwandashan (1♂), Guigang (13♂, 6♀), Lingyun (1♂, 3♀), Longzhou (1♀), Longan (3♂, 3♀), Xingning (1♂, 2♀), Pingxiang(1♂), Qinnan (5♂, 5♀), Rong County (3♂, 2♀), Shanglin (3♂), Tiandeng (1♂, 3♀), Tianyang (1♂), Wanxiu- (1♂, 3♀). **3.** Hainan Province, Changjiang (4♂, 11♀), Chengmai (2♂), Ding’an (1♀), Dongfang (7♂, 5♀), Haikou (2♀), Ledong (6♂, 11♀), Sanya (4♂, 4♀), Tunchang (1♂), Qiongzhong (8♂, 5♀), Wuzhishan (4♂, 7♀). **4.** Fujian Province, Jin’an (2 subadults). **5.** Hong Kong Special Administrative Region (1 subadult). **Vietnam**: Lang Son Province (1♂, 1♀).

#### Description of holotype.

Adult male, medium-sized body, SVL 96.40 mm, trunk length 49.82 mm, head length 24.29 mm, head depth 13.18 mm, head width 15.42 mm, interorbital width 9.47 mm, snout width 5.87 mm, eye-ear length 5.97 mm, eye diameter 8.56 mm, naris-eye length 4.58 mm, jaw width 16.14 mm, snout-eye length 8.82 mm, snout-forelimb length 32.65 mm, tail length 256.15 mm, finger IV length 14.10 mm, toe IV length 19.04 mm, upper arm length 15.80 mm, lower arm length 14.34 mm, upper leg length 21.94 mm, crus length 16.87 mm, forelimb length 46.03 mm, hind limb length 73.17 mm, when hind limbs adpressed forward to reach between eyes and tympanum.

**Table 4.** Morphological data of holotypes and allotypes of *Caloteswangi* sp. nov. Morphometric measurements are in units of mm. For measurement and count methods and abbreviations, see the materials and methods.

Subspecies	*Caloteswangiwangi* ssp. nov.	*C.w.wangi* ssp. nov.	*C.w.hainanensis* ssp. nov.	*C.w.hainanensis* ssp. nov.
Locality	Mt. Daming, Guangxi, China	Mt. Daming, Guangxi, China	Zayun, Hainan, China	Zayun, Hainan, China
specimen type	holotype	allotype	holotype	allotype
Voucher NO.	H2022091534	H2022091533	CIB095629	CIB095630
Sex	♂	♀	♂	♀
Suplab	10:11	9:9	10:10	10:10
Inflab	10:11	9:9	11:11	11:12
NSL	1:1	1:1	1:1	1:1
SoR	3:3	4:4	3:3	4:4
GU	30	27	21	24
VN	48	49	46	50
4FingLm	20:21	21:22	22:22	20:20
4ToeLm	25:25	25:26	27:27	25:26
PtY	3:3	1:1	1:1	1:1
PoS	3:2	2:1	2:2	2:1
DorsalS	42	44	38	43
MidbodyS	42	43	38	37
Eyelid	13:13	12:12	13:13	14:14
SSneck	obliquely upward	obliquely upward	obliquely upward	obliquely upward
Ksneck	modestly to strongly developed	modestly to strongly developed	modestly to strongly developed	modestly to strongly developed
NuchalCrest	significantly larger	significantly larger	significantly larger	significantly larger
DorsalCrests	short, before midbody	short, before midbody	short, before midbody	short, before midbody
SpinesS	1/2	1/3	1/2	1/3
NarEyeS	5	5	5	5
HAF	between eyes and tympanum	between eyes and tympanum	between eyes and tympanum	between eyes and tympanum
TrunkSc	bigger	bigger	bigger	bigger
ParallelR	absence	absence	absence	absence
PostorbitalS	absence	absence	absence	absence
Kstrunk	obliquely upward	obliquely upward	obliquely upward	obliquely upward
GranularS	absent	absent	absent	absent
GP	absent	absent	absent	absent
FS	absent	absent	absent	absent
GF	absent	absent	week present	absent
RSBE	present	present	present	present
TrnkBand	7	7	6	6
LimbBand	7~8	7~8	6~7	7~8
TailBand	22	25	24	23
TrunkSt	2	2	2	2
DorsalBar	absent	week present	absent	week present
HeadL	24.29	23.93	24.47	23.39
HeadH	13.18	13.52	14.41	12.42
HeadW	15.42	14.78	16.08	14.21
Interorb	9.47	10.24	8.51	12.42
EyeEar	5.97	6.17	6.3	5.06
EyeDiam	8.56	7.88	7.66	5.02
JawW	16.14	16.84	16.06	13.47
NarEye	4.58	4.96	4.49	5.69
SnEye	8.82	7.91	7.85	9
SnForeL	32.65	30.4	30.74	25.99
SnW	5.87	5.76	5.29	5.33
SVL	96.4	96.5	88.34	82
TrunkL	49.82	47.02	43.82	40.81
Tail	256.15	243.5	283.89	329
FLL	46.03	41.5	44.82	38.26
HLL	73.17	64.63	71.77	57.97
4FingLng	14.1	13.68	13.41	9.82
4ToeLng	19.04	17.97	21.57	15.36
UparmL	15.8	15.35	16.64	16.36
LoArmL	14.34	15.11	13.59	13.6
UpLegL	21.94	20.76	23.01	17.27
CrusL	16.87	18.08	20.26	17.38

Supralabial scale count 10:11, infralabial scale count 10:11, nasal-supralabial scale rows 1:1, suborbital scale rows 3:3, gular scale count 30, ventral scale count 48, finger IV subdigital lamellae count 20:21, toe IV subdigital lamellae count 25:25, post-tympanic scale count 3:3, post-occipital scale count 3:2, vertebral scales 42, mid-body scale rows 42, dorsal eyelid scales 13:13, scales between anterior chin-shield 1, scales between nasal shield and orbit 5:5, state of scales on side of neck and adjacent shoulder area pointing obliquely upward, keels on these scales are weakly to strongly developed, nuchal and dorsal crest scales short, nuchal crest scales significantly larger than dorsal crest scales, dorsal crest shortens progressively before mid-body, gular fold and fold in front of the shoulder are absent, moderate to large scales in front of forelimb insertion, postorbital spine absent, scales on side of trunk point obliquely upward.

Under stress, the color is khaki (240,230,140) or dark khaki (189,183,107) with pale gray (105,105,105) markings, three black (0,0,0) transverse stripes on top of head, nine blank radial stripes around eyes, throat coloration burly wood (222,184,135) with blank throat stripes, inner-lip coloration is white-smoke (245,245,245), oral cavity coloration is pale flesh (239, 205, 197), tongue coloration is orange (255,165,0), ventral body coloration tan (210,180,140) with dark stripes, presence of dark line on vent midline from throat to pelvis, seven dark khaki bands on dorsum of trunk between axilla and inguen, 7–8 dark khaki fore- and hindlimb cross-bands, striping ventrally on trunk continuous striping, 22 tail cross-bands (the front 9 bands are black and the rear 13 bands are brown [165, 42, 42]).

#### Etymology.

The species name *wangi* is named after Prof. Yuezhao Wang, a former director of the Amphibian and Reptile Research Laboratory (CIB, CAS) and Museum of Herpetology (CIB, CAS) for his research on Chinese herpetology and his contributions in leading the Amphibian and Reptile Research Laboratory through many difficulties. We suggest the English common name Wang’s garden lizard and the Chinese name 中国树蜥 (zhōng guó shù xī).

### ﻿ Calotes wangi hainanensis ssp. nov.

Taxon classificationAnimaliaSquamataAgamidae

6CF7CF9A-EE17-5B6D-B43C-90E42C4B44EE

https://zoobank.org/E7BCDAA5-6A38-4DE4-A44F-62406C7F1F91

Fig. 4 , Table 4

Calotes versicolor Shi et al. 2011 in part; Huang et al. 2013 in part.

#### Type material.

***Holotype*.** Adult male, CIB095629 (filed number HCL200908058). Collected from Chonggongbao Village, Zayun Town, Qiongzhong County, Hainan Province, China (18.9886°N, 109.55716°E, elevation 439 m) by Yong Huang and Bo Cai in August 2009.

***Allotype*.** Adult female, CIB095630 (filed number HCL200908059), with the same locality and collector information as the holotype.

#### Other material examined.

**Hainan Province, China: 1.** Qiongzhong, Wanling Town (2♂, 1♀), Hongmao Town (1♂, 1♀), Zayun Town (1♂), Hongmao Town (3♂, 1♀), Limushan Town (1♀). **2.** Wuzhishan, Hongshan Town (3♂, 2♀), Fanyang Town (5♀). **3.** Dingan, Donghong (1♀). **4.** Ledong, Yongming (1♀). **5.** Tunchang, Dalupo (1♀).

#### Description of holotype.

Adult male, medium-sized body, SVL 88.34 mm. Trunk length 43.82 mm, head length 24.47 mm, head depth 14.41 mm, head width 16.08 mm, interorbital width 8.51 mm, snout width 5.29 mm, eye-ear length 6.30 mm, eye diameter 7.66 mm, naris-eye length 4.49 mm, jaw width 16.06 mm, snout-eye length 7.85 mm, snout-forelimb length 30.74 mm, tail length 283.89 mm, toe IV length 21.57 mm, finger IV length 13.41 mm, upper arm length 16.64 mm, lower arm length 13.59 mm, upper leg length 23.01 mm, Crus length 20.26 mm, forelimb length 44.82 mm, hindlimb length 71.77 mm, when hind limbs are adpressed forward to reach between eyes and tympanum.

Supralabial scale count 10:10, infralabial scale count 11:11, nasal-supralabial scale rows 1:1, suborbital scale rows 3:3, gular scale count 21, ventral scale count 46, finger IV subdigital lamellae count 22:22, toe IV subdigital lamellae count 27:27, post-tympanic scale count 1:1, post-occipital scale count 2:2, vertebral scales 38, mid-body scale rows 38, dorsal eyelid scales 13:13, no scales between anterior chin-shield, scales between nasal shield and orbit 5:5, state of scales on side of neck and adjacent shoulder area pointing obliquely upward, keels on these scales are weakly to strongly developed, nuchal and dorsal crest scales short, nuchal crest scales significantly larger than dorsal crest scales, dorsal crest shortening progressively before mid-body, gular fold and fold in front of the shoulder absent, no patch of granular scales in front of forelimb insertion, pre-axillary area with moderate to large scales, postorbital spine absent, scales on side of trunk pointing obliquely upward.

In ethanol, color is dark khaki or khaki with black or pale gray (105, 105, 105) markings. Tan and black mixed marking on top of head, nine brown (165, 42, 42) radial stripes around eyes, throat coloration burly wood with blank throat stripes, inner lip, oral cavity and tongue coloration is smoky white, ventral body is tan with dark stripes, presence of blank line on vent midline from throat to pelvis, six dark-khaki bands on dorsum of trunk between axilla and inguen, Seven to eight dark-khaki cross-bands on the fore- and hindlimbs, continuous ventral striping on trunk, and 22 cross-bands on tail (anterior nine bands are black, posterior 13 are brown).

#### Etymology.

The specific epithet of *hainanensis* refers to Hainan Island where the new subspecies was discovered. We suggest the English common name Hainan garden lizard and the Chinese name 中国树蜥雷公马亚种 (zhōng guó shù xī léi gōng mǎ yà zhǒng), which comes from a colloquial name for *Caloteswangihainanensis* in Hainan Province, China, meaning Thor’s mount that can predict the weather.

#### Coloration in life.

This species is prone to color changes with different colors during the breeding season and as a result of stress. During the breeding season, adult males are uniformly dark orange (255, 140, 0) to orange on the front half (except for the fingers), with black patches on each side of the neck, darker longitudinal stripes on the chin and darker radial stripes around the eyes. The hind body and toes are uniformly khaki or dark khaki. The dark patches on the sides of the neck are approximately triangular and do not meet, extending from the front of the shoulder to the jaw angles. In the non-breeding season, animals are dark khaki or khaki with black markings.

The coloration of adult females is a relatively pale, uniform khaki over almost the entire body with dark horizontal stripes on a khaki background, and a pale yellow (255, 255, 224) or yellow (255, 255, 0) continuous or discontinuous longitudinal stripe on each side of the trunk in most individuals. They also have black or dark khaki radial stripes around the eyes and dark longitudinal stripes on the chin. Like the females, juveniles are a uniform khaki color over almost the entire body, with dark horizontal stripes and a pair of pale-yellow dorsolateral stripes. Under stress, the coloration of most individuals quickly changes to dark khaki with pale gray markings.

#### Variations.

The means for the measurements of morphological characters are DorsalS 39–52 (average 44.7), Mid-bodyS 36–46 (average 41.2, 2/167 specimen is 35), Eyelid 10–14 (average 12.4, 1/167 specimen is 9 or 15), NarEyeS 4–5 (average 4.9, 6/167 specimen is 6), 4ToeLm 22–27 (average 24.25, 4/167 specimen is 20, 5/167 specimen is 21 and 1/167 specimen is 30), HAF between the eyes and snout (6/167 specimen tympanum), FS absent (5/167 specimen present), SVL 66.14–109.1 mm (average 84.9 mm, 6/187 specimens longer than 100 mm). HAF between the eyes and tympanum (6/167 reaching tympanum, 21/167 reaching eye); FS absent (5/167 present). The ranges for each of these characters are given in Table 5.

**Table 5.** Comparison of morphological characters between *Caloteswangi* sp. nov. and other species in the *C.versicolor* complex. Morphometric measurements are in mm. For measurements, count methods, and abbreviations, see the Materials and methods.

Characters	*Calotesversicolor*	*C.vultuosus*	*C.farooqi*	*C.htunwini*	*C.irawadi*	*C.wangi* sp. nov.
References	Gowande et al. 2021	Gowande et al. 2021	Gowande et al. 2021	Zug et al. 2006	Zug et al. 2006, Liu et al. 2021, this study	this study
Sample Size A	30	20	3	49	16	187
HeadW	0.15~0.28	0.16~0.26	0.19~0.21	/	0.13~0.21(0.18)	0.14~0.21(0.17)
Interorb	0.10~0.14	0.09~0.13	0.12~0.15	/	0.11~0.16(0.14)	0.06~0.17(0.10)
JawW	0.15~0.21	0.17~0.21	0.17~0.20	/	0.14~0.19(0.16)	0.14~0.2(0.17)
HeadH	0.14~0.21	0.16~0.21	0.14~0.16	/	0.13~0.17(0.16)	0.11~0.18(0.15)
SnW	0.05~0.07	0.05~0.08	0.06	/	0.06~0.08(0.06)	0.05~0.08(0.06)
SnEye	0.08~0.11	0.08~0.11	0.09~0.11	/	0.1~0.12(0.11)	0.07~0.14(0.10)
NarEye	0.04~0.07	0.04~0.06	0.04~0.05	/	0.05~0.07(0.06)	0.04~0.1(0.06)
EyeEar	0.06~0.09	0.06~0.09	0.07	/	0.06~0.08(0.07)	0.05~0.1(0.07)
SnForeL	0.34~0.41	0.34~0.45	0.31~0.35	/	0.31~0.39(0.35)	0.25~0.45(0.34)
ForeLimbL	0.48~0.59	0.47~0.59	/	/	0.38~0.7(0.47)	0.41~0.58(0.49)
UparmL	0.14~0.23	0.14~0.23	0.13~0.18	/	0.15~0.21(0.19)	0.13~0.24(0.17)
LoArmL	0.16~0.20	0.16~0.23	0.16~0.17	/	0.14~0.18(0.16)	0.13~0.19(0.16)
HindLimbL	0.69~0.91	0.68~0.85	/	/	0.63~0.8(0.70)	0.64~0.89(0.76)
ForeLimbL/HindLimbL	0.61~0.73	0.65~0.81	/	/	0.58~0.98(0.67)	0.58~0.83(0.65)
UpLegL	0.17~0.25	0.18~0.29	0.21~0.22	/	0.21~0.27(0.24)	0.11~0.32(0.23)
CrusL	0.19~0.28	0.19~0.25	0.22	/	0.21~0.25(0.22)	0.09~0.3(0.21)
4ToeLng	0.15~0.26	0.15~0.23	0.13~0.20	/	0.17~0.21(0.19)	0.08~0.3(0.21)
4FingLng	0.10~0.18	0.10~0.15	0.12~0.13	/	0.09~0.15(0.12)	0.11~0.21(0.14)
TrunkL	0.38~0.50	0.37~0.50	0.48~0.52	/	0.45~0.61(0.51)	30.44~51.95(44.24)
Sample Size B	30	20	3	49	76	167
SVL (adult means)	>90 mm	>90 mm	>90 mm	<90 mm	<90 mm	<90 mm
TailL (adult means)	268.2	/	/	150.8	240.5	242.4
CanthR	6~9	7~8	8	8	5~7(6.11)	4~5(4.91)
VertS	31~51	35~62	40~44	38~57 (47.3)	36~59 (48.2)	39~52(44.7)
Midbody	36~46	37~45	41~44	39~53 (47.1)	38~51(44.9)	36~46(41.2)
4ToeLm	21~30	23~28	24~25	18~26 (22.7)	22~29(24.6)	19~27(24.2)
Eyelid	10~15(12)	11~14 (12)	9~11 (10)	12~13(11.2)	12~13(12.8)	10~14(12.4)
SSstrunk	obliquely upward	obliquely upward	obliquely upward	obliquely upward	obliquely upward	obliquely upward
SSforelimb	no patch of granular scales	no patch of granular scales	no patch of granular scales	no patch of granular scales	no patch of granular scales	no patch of granular scales
SSneck	obliquely upward	obliquely upward	obliquely upward	horizontal	obliquely upward	obliquely upward
Keels	weakly to strongly developed	weakly to strongly developed	weakly to strongly developed	modestly to strongly developed	weakly to strongly developed	weakly to strongly developed
NucSpot	approximately bean-shaped, not extending to the lower jaw	extending from the front of the shoulder to the ½ jaw	extending from the front of the shoulder to the ¾ jaw	frequently in females, not extended to the jaw	frequently in females, few extended to the jaw	approximately triangular, extending from the front of the shoulder to the jaw angles
CTG	pale flesh color	/	/	/	/	orange
DorsalCrests	long, shortens progressively to the base of the tail	short, shortens progressively to the mid-body;	short, shortens progressively to the mid-body	short, shortens progressively to the mid-body	short, shortens progressively after the mid-body	short, shortens progressively before the mid-body
NuchalCrests	long, distinct larger	short, distinct larger	short, distinct larger	short, slightly larger	short, distinct larger	short, distinct larger
SpinesS	long, > 2/3 tympanum diameter	short, < 1/2 tympanum diameter	short, < 1/2 tympanum diameter	short, < 1/2 tympanum diameter	short, < 1/3 tympanum diameter	short, < 1/2 tympanum diameter
NarEyeS	> 6	> 6	< 6	/	5~7(6)	4~5(4.9)
HAF	/	/	/	/	reaching tympanum	crossing tympanum
FS	absence	absence	absence	absence	absence	absence

*Caloteswangiwangi* ssp. nov. can be separated from *C.w.hainanensis* ssp. nov. by the following characters: **1.**Eyelid 10–13 (average 12.4, few 9 and 15) vs 13–14 (average 13, few 12. **2.**Tail/SVL 1.59–3.36 (average 2.83) vs 1.77–4.01 (average 3.15). **3.** Male, UpLegL/SVL 0.11–0.26 (average 0.23) vs 0.23–0.32 (average 0.25). **4.** Male, CrusL/SVL 0.09–0.24 (average 0.21) vs 0.21–0.3 (average 0.24).

#### Diagnosis.

*Caloteswangi* sp. nov. can be separated from all other species of *Calotes* by having the following characters: **1.** Medium-sized adult male, SVL < 90 mm (66.86–109.1 mm, average 85.64 mm). **2.** Smaller HeadW in males, 11.64–19.72 (average 14.84). **3.** Larger HindLimbL/SVL 0.64–0.89 (average 0.75). **4.** Larger 4ToeLng/SVL 0.08–0.3 (average 0.21). **5.** Larger 4FingLng/SVL 0.11–0.21 (average 0.14). **6.**Eyelid 10–14 (average 12.4). **7.**NarEyeS 4–5 (average 4.9). **8.** Scales on side of trunk point obliquely upward. **9.** Patch of granular scales in front of forelimb insertion absent. **10.** Scales on side of neck and adjacent shoulder area point obliquely upward; keels on neck scales are weakly to strongly developed. **11.** Paired dark patches are approximately triangular, extending from the front of the shoulder to the jaw angles. **12.** Coloration of tongue is orange. **13.** Nuchal and dorsal crest scales short; dorsal crest shortening progressively before mid-body; nuchal crest distinctly larger than dorsal crest. **14.** Supratympanic spines are short. **15.** Fourth toe with claw can stretch between the eyes and tympanum when hind limbs are adpressed forward. **16.** Fold in front of the shoulder is absent. **17.** During breeding season, males are uniformly dark orange to orange on the front half (except for the fingers) with black patches on both sides of neck and darker radial stripes around eyes; Hind body and toes a uniform khaki or dark khaki.

#### Comparisons.

*Caloteswangi* sp. nov. was previously recognized as *C.versicolor* by previous authors but it can easily be distinguished from true *C.versicolor* (India) by adult males having shorter average SVL < 90 mm vs > 90 mm in *C.versicolor*; fewer scales between the nasal shield and the orbit 4–5 (average 5), < 6 vs > 6; dark patches on neck extending to jowl vs not extending to jowl; nuchal and dorsal crest scales short and nuchal crest larger vs nuchal and dorsal crest scales well developed and nuchal crest scales only slightly larger than dorsal crest scales; pair of short supratympanic spines on each side of the head vs two well-separated supratympanic spines; coloration of tongue orange vs pale flesh color (239, 205, 197).

This species can be separated from others in the *C.versicolor* complex by the following characters (Zug et al. 2006): 36–46 mid-body scale rows (average 41.2) vs 45–55 in the Nat-Ma-Taung *versicolor* group and 46–53 in the Moyingyi *versicolor* group; forearm stripe and paired nuchal spots present in adult vs absent in the Thai-east *versicolor* group.

The new species differs from *Calotesirawadi* by its smaller HeadW in males, 11.64–19.72 vs 13.08–28.3 in *C.irawadi*, shorter Interorb 4.59–12.89 vs 6.9–15.07; longer 4FingLng/SVL 0.11–0.21 vs 0.09–0.15; fewer mid-body scale rows 36–46 (average 41.2) vs 38–51 (average 44.9); fewer scales between the nasal shield and the orbit 4–5 (average 4.9) vs 5–7 (average 6.1); progressively shortened dorsal crest before mid-body vs progressively shortened after mid-body (especially in males), fourth toe with claw reaching between the eyes and tympanum when hind limbs adpressed forward vs reaching tympanum.

The new species can be separated from *Caloteshtunwini* Zug & Vindum, 2006 (Gowande et al. 2021) by the scales on the side of the neck and adjacent shoulder area pointing obliquely upward vs horizontally in *C.htunwini*; the keels on these scales weakly to strongly developed vs modestly to strongly developed. The new species can be separated from from *C.farooqi* Auffenberg & Rehmann, 1995 by the shorter average adult male SVL, < 90 mm vs > 90 mm in *C.farooqi*; the smaller HeadW/SVL, 0.14–0.21 vs 0.19–0.21; more dorsal eyelid scales, 10–14(average 12.4) vs 9–11 (average 10) in *C.farooqi*. The new species can be separated from *C.vultuosus* (Harlan, 1825) by shorter average adult male SVL, < 90 mm vs > 90 mm in *C.vultuosus*; smaller HeadW/SVL, 0.14–0.21 vs 0.16–0.2; smaller HeadH/SVL, 0.11–0.18 vs 0.16–0.21; more scales between the nasal shield and the orbit, 4–5 (average 4.9) vs > 6; dark patches on neck not extending to 1/2 jowl vs stretching to 1/2 jowl in *C.vultuosus*.

This species can be separated from other members of *Calotes* by a combination of the following characters: **1.** Crescent-shaped patch of granular scales absent at the forelimb insertion vs present in *C.emma*, *C.grandisquamis* Günther, 1875, *C.jerdoni* Günther, 1870, *C.mystaceus* Duméril & Bibron, 1837, and *C.nemoricola* Jerdon, 1853. **2.** Keeled dorsal scales vs smooth in *C.medogensis* Zhao & Li, 1984. **3.** Mid-body scale rows 36–46 (average 41.2) vs 49–65 in *C.emma*, 27–35 in *C.grandisquamis*, 45–57 in *C.jerdoni*, 58–63 in *C.maria*, 48–60 in *C.minor*, 45–58 in *C.mystaceus*. **4.** Nuchal and dorsal crest scales short, nuchal crest distinctly larger vs nuchal spines much longer, dorsal spines reduced in *C.maria* and *C.nemoricola*. **5.** Pair of short supratympanic spines on each side of the head vs a row of three or four compressed supratympanic spines in *C.grandisquamis* and *C.nemoricola*, eight or nine compressed spines above the tympanum in *C.calotes* (Linnaeus, 1758), two parallel rows of supratympanic scales in *C.jerdoni* and *C.maria* Gray, 1845, and a single well-developed postorbital spine in *C.emma*. **6.** Homogeneous scalation on the dorsal region and a comparatively well-developed dorsal crest vs heterogeneous scalation and an undeveloped dorsal crest in *C.paulus* Smith, 1935 and *C.zolaiking* Giri, Chaitanya, Mahony, Lalronunga, Lakrinchhana, Das, Sarkar, Karanth & Deepak, 2019. **7.** Concave orbital region and absence of row of erect scales on sides of neck vs no concave orbital region and presence of row of erect scales in *C.bhutanensis* Biswas, 1975. **8.** Absence of fold in front of shoulder vs presence of fold in *C.chincollium* Vindum, 2003, *C.nigriplicatus* Hallermann, 2000, *C.bachae* Hartmann, Geissler, Poyarkov, Ihlow, Galoyan, Rödder & Böhme, 2013, *C.geissleri* Wagner, Ihlow, Hartmann, Flecks, Schmitz & Böhme, 2021, *C.goetzi* Wagner, Ihlow, Hartmann, Flecks, Schmitz & Böhme, 2021, *C.mystaceus* and *C.vindumbarbatus* Wagner, Ihlow, Hartmann, Flecks, Schmitz & Böhme, 2021. **9.** Posterodorsal orientation of lateral body scales and absence of shoulder pit vs posteroventral orientation and presence of shoulder pit in *C.ceylonensis* Müller, 1887, *C.desilvai* Bahir & Maduwage, 2005, *C.liocephalus* Günther, 1872, *C.liolepis* Boulenger, 1885, *C.manamendrai* Amarasinghe & Karunarathna, 2014, *C.nigilabris* Peters, 1860, *C.pethiyagodai* Amarasinghe, Karunarathna & Hallermann, 2014.

Measurements and scale counts of specimens are given in Table 5 and Suppl. material 1: table S2.

#### Distribution and natural history.

*Caloteswangi* sp. nov. is a transboundary species, ranging from southern China (Fujian Province, Guangdong Province, Hainan Province and Guangxi Zhuang Autono­mous Region, southern Hunan Province , Macao, and Hong Kong) to northern Vietnam (Lang Son). The eastern Yunnan region in China is also likely to harbor this species (Yang and Rao 2008; Shuo Liu, pers. comm. 17 April 2023). The known altitude range of *C.w.wangi* ssp. nov. is 15~2000 m and *C.w.hainanensis* is 47~1867 m. It is found in subtropical evergreen broad-leaved forests and tropical monsoon forests in southern China and northern Vietnam, mostly in mountainous areas, hills and plains on forest edges, arable land, shrub lands, and even urban green belts. It is active at the edge of the forest, and when it is in danger, it rushes into bushes or climbs tree trunks to hide. It is active from April to October every year, while in the tropics it is active from March to November or even longer. The authors investigated in Hainan and found that *C.wangi* sp. nov. was active from 7:30~11:30 hrs in summer and 15:00~19:00 hrs in the afternoon. Investigations in Guangxi found that the lizards lie on sloping shrub branches at night, sleeping close to the branches.

Both *Caloteswangi* sp. nov. and *C.irawadi* are oviparous. It has been reported that females of *C.wangi* sp. nov. contained 5–12 (17 in one specimen in Mt. Diaoluo, Hainan) mature eggs from late April to September, while females of *C.irawadi* contained 12 mature eggs in June (Zhao et al. 1999). After our dissections, 13 specimens of *C.wangi* sp. nov. from Hainan obtained in August had 5–8 eggs (average 6), and five *C.irawadi* from Lushui, Yunnan obtained in late May were pregnant with 5–15 eggs (average 11).

*Caloteswangi* sp. nov. eats a variety of insects, spiders, and other arthropods (Zhao et al. 1999).

#### Specimens examined.

*Calotesirawadi* (*n* = 18). **Myanmar**: Kawnglanghpu (CIB097515). **China**: Yunnan Province, Lushui City (Shangjiang CIB001824-26), Baoshan City (Shangjiang CIB001819-23, CIB001827-28), Dehong Dai and Jingpo Autonomous Prefecture (Hongbenghe CIB116106 and CIB116212, Tongbiguan KIZ 059191, KIZ NB20180905, KIZ HBH20200913 and KIZ HBH20200914), Xishuangbanna Dai Autonomous Prefecture (CIB001610).

### ﻿Key to the species of *Calotes* in China

Modified from Zhao et al. (1999), Zug et al. (2006), Gowande et al. (2016), Pal et al. (2018), Giri et al. (2019), Gowande et al. (2021), and Wagner et al. (2021).

**Table:** 

1	Presence of heterogeneous scales on dorsum, caudal vertebrae with transverse processes 14	***Calotespaulus***
–	Absence of heterogeneous scales on dorsum	**2**
2	Smooth dorsal scales	***C.medogensis***
–	Keeled dorsal scales	**3**
3	Post orbital spine present	***C.emma***
–	Post orbital spine absent	**4**
4	Two parallel rows of compressed spines above tympanum, dorsum green	***C.jerdoni***
–	No parallel rows of compressed scales above tympanum, dorsum not green	**5**
5	Presence of fold in front of the shoulder	**6**
–	Absence of fold in front of the shoulder	**7**
6	No brownish dorsolateral blotches, whitish stripe from tip of snout continuing to beyond limb insertion	***C.vindumbarbatus***
–	Prominent dark brown dorsolateral blotches	***C.goetzi***
7	Fewer scales (4 or 5, average 4.9) between the nasal shield and the orbit, fourth toe with claw crossing tympanum when hind limbs adpressed forward	**8**
–	More scales (5–7, average 6.1) between the nasal shield and the orbit, fourth toe with claw reaching tympanum when hind limbs adpressed forward	***C.irawadi***
8	Fewer eyelid scales (10–13, average 12.4), Tail/SVL (1.59–3.36, average 2.83), male UpLegL/SVL (0.11–0.26, average 0.23), male CrusL/SVL (0.09–0.24, average 0.21)	***C.w.wangi* ssp. nov.**
–	More eyelid scales (13–14, average 13), Tail/SVL (1.77–4.01, average 3.15), male UpLegL/SVL (0.23–0.32, average 0.25), male CrusL/SVL (0.21–0.3, average 0.24)	***C.w.hainanensis* ssp. nov.**

## ﻿Discussion

Based on extensive spatial sampling across China and comprehensive data on morphology and genetics for species delimitation, we add a new species and more stability to the systematics of *Calotes*. Another interesting finding from this study is the presence of subspecies, which highlights the need for more thorough geographic sampling to uncover cryptic lineages. This study provides additional data to clarify the taxonomic status of the widely distributed *Calotesversicolor* complex. *Caloteswangi* sp. nov. is described as a new species in the Guangdong, Guangxi, Hainan, Hong Kong, and Fujian populations of *C.versicolor* complex in this study.

The Yunnan populations in the *Calotesversicolor* complex were recorded from Wenshan Prefecture (Funing), Dehong Prefecture (Yingjiang and Longchuan), Nujiang Prefecture (Lushui), Baoshan City, Dali Prefecture (Yangbi), Pu’er City (Menglian), and Xishuangbanna Prefecture (Zhao et al. 1999; Yang and Rao 2008). Liu et al. (2021) confirmed that the *C.versicolor* complex in Dehong Prefecture contains *C.irawadi*, but the records from Lushui and Baoshan were confused regarding the specific collection localities. The CIB collection information only covered Lushui, while the old labels on the bottles of Zhao and Yang (1997) indicated that the samples were from Lushui and Baoshan. According to our study, the morphology of that group of specimens was consistent with *C.irawadi*. One sequence we obtained from Lushui and the Baoshan region (Mt. Gaoligong) was in the same clade in the phylogenetic tree as *C.irawadi* from the type locality (Fig. 2); therefore, we conclude that the records from Baosan and Lushui refer to *C.irawadi*. In the records of Xishuangbanna, Zeng (1994) recorded an individual from the Xishuangbanna Tropical Botanical Garden, in which the morphology was consistent with *C.irawadi*; however, other records were not detailed, such as CIB001610. One sequence we obtained from Xishuangbanna is also in the same clade in the phylogenetic tree as *C.irawadi* from the type locality; therefore, we can also state that the records from Xishuangbanna likely refer to *C.irawadi*. The record of Menglian lacks specimen information. Menglian is close and has an environment like that of the Min-Gon-Taung Wildlife Sanctuary in Myanmar, one of the distribution sites of *C.irawadi* (Zug et al., 2006). Whether this record refers to *C.irawadi* needs further study. The record of Yangbi County in northern Yunnan also lacks specimen information (Yang and Rao 2008). Yangbi is near to and the environment is similar to that of Lushui and Baoshan. Whether this record belongs to *C.irawadi* remains unclear. Funing County is in eastern Yunnan, and its topography and climate are like those in western Guangxi. Liu et al. (2021) found that the populations of *C.versicolor* complex in eastern and western Yunnan contained different species during their surveys. The morphology of the eastern population was similar to that of the Guangdong and Guangxi populations, and it is likely to be *C.wangi* sp. nov. (Shuo Liu, pers. comm. 17 April 2023). Thus, the eastern Yunnan populations of *C.versicolor* complexes consist of *C.irawadi* (the records of Meng Lian and Yangbi need further study), while western Yunnan is most likely to have *C.wangi* sp. nov.

The Hunan population of the *Calotesversicolor* complex was recorded from Yizhang County (Deng and Ye 1996, 1997). After comparing the morphological data with the new species in the article, we conclude that the record in Hunan represents *C.wangi* sp. nov.

The literature pertaining to the *Calotesversicolor* complex in Macau also lacks morphological or molecular information. By referring to the morphology in the photographs provided by Hoi Yan Wong from the Macau population of *C.versicolor*, as well as the distance and climatic environment between Macau and the Hong Kong, Guangdong Province, we consider that the Macau population is also *C.wangi* sp. nov.

Gowande et al. (2021) mentioned a single sequence of *Calotesirawadi* from Huanan Province, China (locality 73). The sequence was HM362986 (Xm 3490), which was uploaded to GenBank by Gu et al. (2011) together with HM362984 and HM362985. The location of these specimens was the Huanan district of China (i.e., southern China), not Huanan Province. After phylogenetic analysis, these three sequences were clustered together in the clade of *C.wangi* sp. nov., and the morphological characters of these specimens were also consistent with this species.

To date, there are nine species of tree lizards in China: *Calotesemma*, *C.jerdoni*, *C.medogensis*, *C.paulus* (Cai et al. 2022a), *C.irawadi* (Liu et al. 2021), *C.goetzi* (Wagner et al. 2021), and *C.vindumbarbatus* (Liu et al. 2022) and *C.wangi* sp. nov. (this study). *Calotesversicolor* is not distributed in China. In the field surveys, we found that the populations of *C.wangi* sp. nov. were relatively large, and the species is not threatened at present. However, in some areas, their habitat was being fragmented. In addition, their bodies are used medicinally and the lizards are also eaten. We suggest that the local government strengthen the protection of their ecological environment and pay close attention to the population dynamics.

## Supplementary Material

XML Treatment for Calotes wangi

XML Treatment for Calotes wangi hainanensis

## ﻿Additional information

### Author ORCIDs

Yong Huang https://orcid.org/0000-0002-3493-9468

Bo Cai https://orcid.org/0000-0002-4214-9060

### Data availability

All of the data that support the findings of this study are available in the main text or Supplementary Information.

## Supplementary materials

Supplementary material 1 Supplementary information Data type docx Explanation note **table S1.** Voucher specimens and GenBank accession numbers of DNA sequences of *Caloteswangi* sp. nov., and other species in the genus *Calotes* used in this study. **table S2.** Comparison of measurement data between different populations of *Caloteswangi* sp. nov. and C.cf.versicolor populations. **fig. S1.** Local enlarged phylogenetic tree structure corresponding to *Caloteswangihainanensis* ssp. nov. (Lineage A) and *C.w.wangi* ssp. nov. (Lineage B), respectively. https://binary.pensoft.net/file/955476This dataset is made available under the Open Database License (http://opendatacommons.org/licenses/odbl/1.0/). The Open Database License (ODbL) is a license agreement intended to allow users to freely share, modify, and use this Dataset while maintaining this same freedom for others, provided that the original source and author(s) are credited.Yong Huang, Hongyu Li, Yilin Wang, Maojin Li, Mian Hou, Bo Cai
