# Supplementary material for: ﻿Taxonomic review of the Calotesversicolor complex (Agamidae, Sauria, Squamata) in China, with description of a new species and subspecies
Source: Zookeys. 2023 Dec 20;1187:63–89. doi: 10.3897/zookeys.1187.110704 (PMC10755893; doi:10.3897/zookeys.1187.110704)

**Table S1** Voucher specimens and GenBank accession numbers of DNA sequences of *Calotes wangi* sp. nov., and other species in the genus *Calotes* used in this study.

| ID | Species | Subspecies | Locality (Abbreviation) | Voucher NO. | GenBank | Population number | Latitude | Longitude | References |
| --- | --- | --- | --- | --- | --- | --- | --- | --- | --- |
| 1 | *Calotes wangi* sp. nov. | *C. w. wangi* ssp. nov. | Mt. Daming, Guangxi, China (DM) | 2022091533~2022091541 | OR828811~  OR828819 | 9 | 23.548581 | 108.353307 | This study |
| 2 | *C. wangi* sp. nov. | *C. w. wangi* ssp. nov. | Taohua, Guangxi, China (TH) | 201606139~201606142 | OR828796~  OR828798 | 3 | 24.212539 | 106.611948 | This study |
| 3 | *C. wangi* sp. nov. | *C. w. wangi* ssp. nov. | Bama, Guangxi, China (BM) | 201606144~201606148 | OR828799~  OR828803 | 5 | 24.09138 | 107.250248 | This study |
| 4 | *C. wangi* sp. nov. | *C. w. wangi* ssp. nov. | Naheng, Guangxi, China (NH) | 201606143 | OR828804 | 1 | 23.953857 | 107.065068 | This study |
| 5 | *C. wangi* sp. nov. | *C. w. wangi* ssp. nov. | Fucheng, Guangxi, China (FC) | 201604082~201604087 | OR828805~  OR828810 | 6 | 23.391183 | 108.247992 | This study |
| 6 | *C. wangi* sp. nov. | *C. w. wangi* ssp. nov. | Nanning, Guangxi, China (NN) | HC201002279~HC201002281 | KC87576, KC875761, KC875763 | 3 | 22.86 | 108.37 | Huang et al. 2013 |
| 7 | *C. wangi* sp. nov. | *C. w. wangi* ssp. nov. | Wutang, Guangxi, China (WT) | 201511048-201511050,201511052 | OR828820~OR828823 | 4 | 22.945312 | 108.555563 | This study |
| 8 | *C. wangi* sp. nov. | *C. w. wangi* ssp. nov. | Dingdang, Guangxi, China (DD) | 201604088~201604102 | OR828824~OR828838 | 15 | 23.13039 | 107.976043 | This study |
| 9 | *C. wangi* sp. nov. | *C. w. wangi* ssp. nov. | Gangbei, Guangxi, China (GB) | 201606104~201606108 | OR828839~OR828843 | 5 | 23.093115 | 109.540017 | This study |
| 10 | *C. wangi* sp. nov. | *C. w. wangi* ssp. nov. | Wuzhou, Guangxi, China (WZ) | 201606134~201606137 | OR828844~OR828847 | 4 | 23.526721 | 111.329018 | This study |
| 11 | *C. wangi* sp. nov. | *C. w. wangi* ssp. nov. | Cenxi, Guangxi, China (CX) | 201606130~201606132 | OR828848~OR828850 | 3 | 22.914898 | 110.958258 | This study |
| 12 | *C. wangi* sp. nov. | *C. w. wangi* ssp. nov. | Rongxi, Guangxi, China (RX) | 201606115~201606119 | OR828851~OR828855 | 5 | 22.784373 | 110.43628 | This study |
| 13 | *C. wangi* sp. nov. | *C. w. wangi* ssp. nov. | Qinnanqu, Guangxi, China (QN) | 201510030-201510033,201510035 | OR828856~OR828860 | 5 | 21.980953 | 108.653817 | This study |
| 14 | *C. wangi* sp. nov. | *C. w. wangi* ssp. nov. | Wenming, Guangxi, China (WM） | 201512053,201512056 | OR828861~OR828862 | 2 | 22.4119 | 109.6998 | This study |
| 15 | *C. wangi* sp. nov. | *C. w. wangi* ssp. nov. | Fangchenggang, Guangxi, China (FCG) | 201510039-201510044,201510047 | OR828863~OR828869 | 7 | 21.635534 | 108.301372 | This study |
| 16 | *C. wangi* sp. nov. | *C. w. wangi* ssp. nov. | Yinhaiqu, Guangxi, China (YH) | 201509019~201509020,201509022~201509025 | OR828870~OR828875 | 6 | 21.468197 | 109.078404 | This study |
| 17 | *C. wangi* sp. nov. | *C. w. wangi* ssp. nov. | Weizhoudao, Guangxi, China (WZD) | 201509003~201509007,201509009,201509011,201509012,201509014~201509017,201509027,201509028 | OR822208～OR822221 | 14 | 21.066718 | 109.139317 | This study |
| 18 | *C. wangi* sp. nov. | *C. w. wangi* ssp. nov. | Fuchao, Guangdong, China (FCC) | 201606125~201606129 | OR828876~OR828879 | 4 | 22.781087 | 111.608735 | This study |
| 19 | *C. wangi* sp. nov. | *C. w. wangi* ssp. nov. | Xinyi, Guangdong, China (XY) | 201606120~201606123 | OR828880~OR828883 | 4 | 22.339085 | 110.937615 | This study |
| 20 | *C. wangi* sp. nov. | *C. w. wangi* ssp. nov. | Yangchun, Guangdong, China (YC) | 201606109~201606114 | OR828884~OR828889 | 6 | 22.141142 | 111.78446 | This study |
| 21 | *C. wangi* sp. nov. | *C. w. wangi* ssp. nov. | Haian, Guangdong, China (HA) | HC200908192~HC200908199, HCL200908276 | KC875759，KC875749~KC875756 | 9 | 20.28 | 110.21 | Huang et al. 2013 |
| 22 | *C. wangi* sp. nov. | *C. w. wangi* ssp. nov. | Lang Son, Vietnam (LS) | HC201006282~HC201006288 | KC875765~KC87577 | 7 | 22.15 | 106.65 | Huang et al 2013 |
| 23 | *C. wangi* sp. nov. | *C. w. wangi* ssp. nov. | Lianjiang, Fujian, China (LJ) | / | / |  | 26.2189 | 119.4314 | Hu et al. 2022 |
| 24 | *C. wangi* sp. nov. | *C. w. wangi* ssp. nov. | Jin’an, Fujian, China (JA) | 2022091526-2022091527 | OR878647~OR878648 | 2 | 26.175443 | 119.296059 | This study |
| 25 | *C. wangi* sp. nov. | *C. w. wangi* ssp. nov. | Hongkong, China (HK) | HC201006295 | KC875772 | 1 | 22.4 | 114.11 | Huang et al. 2013 |
| 26 | *C. wangi* sp. nov. | *C. w. hainanensis* ssp. nov. | Tunchang, Hainan, China (TC) | HCL200907005~HCL200907007 | KC875611~KC875613 | 3 | 19.58298 | 110.17577 | Huang et al. 2013 |
| 27 | *C. wangi* sp. nov. | *C. w. hainanensis* ssp. nov. | Wanling, Hainan, China (WL) | HCL200907047~HCL200907052 | KC875614~KC875619 | 6 | 19.13316 | 109.90797 | Huang et al. 2013 |
| 28 | *C. wangi* sp. nov. | *C. w. hainanensis* ssp. nov. | Jiachai, Hainan, China (JC) | HCL200907053~HCL200907054 | KC875620~KC875621 | 2 | 19.04 | 109.79 | Huang et al. 2013 |
| 29 | *C. wangi* sp. nov. | *C. w. hainanensis* ssp. nov. | Hongmao, Hainan, China (HM) | HCL200907055，HCL200908071 | KC875622，KC875638 | 2 | 19.03 | 109.68 | Huang et al. 2013 |
| 30 | *C. wangi* sp. nov. | *C. w. hainanensis* ssp. nov. | Chonggongbao, Hainan, China (CG) | HCL200908056~HCL200908061 | KC875623~KC875628 | 6 | 18.9886 | 109.55716 | Huang et al. 2013 |
| 31 | *C. wangi* sp. nov. | *C. w. hainanensis* ssp. nov. | Fanxiang, Hainan, China (FX) | HCL200908062~HCL200908068 | KC875629~KC875635 | 7 | 19.03 | 109.67 | Huang et al. 2013 |
| 32 | *C. wangi* sp. nov. | *C. w. hainanensis* ssp. nov. | Zayun, Hainan, China (SY) | HCL200908069 | KC875636 | 1 | 19.02 | 109.57 | Huang et al. 2013 |
| 33 | *C. wangi* sp. nov. | *C. w. hainanensis* ssp. nov. | Maoyang, Hainan, China (MY) | HCL200908070 | KC875637 | 1 | 18.91 | 109.51 | Huang et al. 2013 |
| 34 | *C. wangi* sp. nov. | *C. w. hainanensis* ssp. nov. | Hela, Hainan, China (HL) | HCL200908072~HCL200908075 | KC875639~KC875642 | 4 | 19 | 109.67 | Huang et al. 2013 |
| 35 | *C. wangi* sp. nov. | *C. w. hainanensis* ssp. nov. | Hongshan, Hainan, China (HS) | HCL200908076~HCL200908082 | KC875643~KC875649 | 7 | 18.86 | 109.53 | Huang et al. 2013 |
| 36 | *C. wangi* sp. nov. | *C. w. hainanensis* ssp. nov. | Fanyang, Hainan, China (FY) | HCL200908083~HCL200908089 | KC875650~KC875656 | 7 | 18.88 | 109.36 | Huang et al. 2013 |
| 37 | *C. wangi* sp. nov. | *C. w. hainanensis* ssp. nov. | Limushan, Hainan, China (LMS) | HCL200908168~HCL200908172 | KC875731~ KC875735 | 5 | 19.22 | 109.81 | Huang et al. 2013 |
| 38 | *C. wangi* sp. nov. | *C. w. hainanensis* ssp. nov. | Huangzhu, Hainan, China (HZ) | HCL200908181~HCL200908184 | KC875741~ KC875744 | 4 | 19.44 | 110.45 | Huang et al. 2013 |
| 39 | *C. wangi* sp. nov. | *C. w. hainanensis* ssp. nov. | Fuwen, Hainan, China (FW) | CIB091425~CIB091426 | KC875778，KC875777 | 2 | 19.55 | 110.26 | Huang et al. 2013 |
| 40 | *C. wangi* sp. nov. | *C. w. hainanensis* ssp. nov. | Lingshui, Hainan, China (LS) | CIB91435~CIB91452 | KC875787~ KC875804 | 18 | 18.71 | 109.95 | Huang et al. 2013 |
| 41 | *C. wangi* sp. nov. | *C. w. wangi* ssp. nov. | Wenchang, Hainan, China (WC) | HC200907002 | KC875610 | 1 | 19.86 | 110.6 | Huang et al. 2013 |
| 42 | *C. wangi* sp. nov. | *C. w. wangi* ssp. nov. | Datian, Hainan, China (DT) | HCL200908091~HCL200908092，HCL200908094~HCL200908098 | KC875657~KC875663 | 7 | 19.12 | 108.83 | Huang et al. 2013 |
| 43 | *C. wangi* sp. nov. | *C. w. wangi* ssp. nov. | Donghe, Hainan, China (DH) | HCL200908099~HCL200908104 | KC875664~KC875669 | 6 | 19.02 | 108.99 | Huang et al. 2013 |
| 44 | *C. wangi* sp. nov. | *C. w. wangi* ssp. nov. | Wanting, Hainan, China (WT) | HCL200908105~HCL200908109 | KC875670~KC875674 | 5 | 19.12 | 109.08 | Huang et al. 2013 |
| 45 | *C. wangi* sp. nov. | *C. w. wangi* ssp. nov. | Sanpai, Hainan, China (SP) | HCL200908110~HCL200908121, HCL200908200, HC200908278 | KC875675~KC875760 | 13 | 19.01 | 109.14 | Huang et al. 2013 |
| 46 | *C. wangi* sp. nov. | *C. w. wangi* ssp. nov. | Bawangling, Hainan, China (BWL) | HCL200908122~HCL200908127 | KC875687~KC87569 | 6 | 19.03 | 109.12 | Huang et al. 2013 |
| 47 | *C. wangi* sp. nov. | *C. w. wangi* ssp. nov. | Jianfeng, Hainan, China (JF) | HCL200908128~HCL200908134 | KC875693~KC875699 | 7 | 18.7 | 108.81 | Huang et al. 2013 |
| 48 | *C. wangi* sp. nov. | *C. w. wangi* ssp. nov. | Tianya, Hainan, China (TY) | HCL200908136~HCL200908139, HCL200908141~HCL200908146, HCL200908275 | KC875758, KC875708~KC875709, KC8757095~KC8757097, KC8757092, KC875700~ KC875704 | 11 | 18.31 | 109.27 | Huang et al. 2013 |
| 49 | *C. wangi* sp. nov. | *C. w. wangi* ssp. nov. | Zhizhong, Hainan, China (ZZ) | HCL200908147~HCL200908153 | KC875710~KC875716 | 7 | 18.63 | 109.29 | Huang et al. 2013 |
| 50 | *C. wangi* sp. nov. | *C. w. wangi* ssp. nov. | Jiangbian, Hainan, China (JB) | HCL200908154~HCL200908155 | KC875717~KC875718 | 2 | 18.82 | 109.06 | Huang et al. 2013 |
| 51 | *C. wangi* sp. nov. | *C. w. wangi* ssp. nov. | Yongming, Hainan, China (YM) | HCL200908156~HCL200908159 | KC875719~KC875722 | 4 | 18.77 | 109.17 | Huang et al. 2013 |
| 52 | *C. wangi* sp. nov. | *C. w. wangi* ssp. nov. | Zhiwei, Hainan, China (ZW) | HCL200908160~HCL200908167 | KC875723~ KC875730 | 8 | 18.76 | 109.08 | Huang et al. 2013 |
| 53 | *C. wangi* sp. nov. | *C. w. wangi* ssp. nov. | Fushan, Hainan, China (FS) | HCL200908173~HCL200908178 | KC875736~KC875740 | 5 | 19.87 | 109.92 | Huang et al. 2013 |
| 54 | *C. wangi* sp. nov. | *C. w. wangi* ssp. nov. | Haikou, Hainan, China (HK) | HC200907001, HCL200908185~HCL200908191 | KC875745~KC875748, KC875609F | 5 | 20 | 110.34 | Huang et al. 2013 |
| 55 | *C. wangi* sp. nov. | *C. w. wangi* ssp. nov. | Yanfeng1, Hainan, China (YF1) | CIB091420 | KC875773 | 1 | 19.95 | 110.55 | Huang et al. 2013 |
| 56 | *C. wangi* sp. nov. | *C. w. wangi* ssp. nov. | Yanfeng2, Hainan, China (YF2) | CIB091421, CIB091423 | KC875774~ KC875775 | 2 | 19.96 | 110.56 | Huang et al. 2013 |
| 57 | *C. wangi* sp. nov. | *C. w. wangi* ssp. nov. | Changliu, Hainan, China (CL) | CIB091424 | KC875776 | 1 | 20.03 | 110.16 | Huang et al. 2013 |
| 58 | *C. wangi* sp. nov. | *C. w. wangi* ssp. nov. | Nanfeng, Hainan, China (NF) | CIB091427~CIB091433 | KC875779~KC875785 | 7 | 19.4 | 109.56 | Huang et al. 2013 |
| 59 | *C. wangi* sp. nov. | *C. w. wangi* ssp. nov. | Nada1, Hainan, China (ND1) | CIB091434 | KC875786 | 1 | 19.5 | 109.56 | Huang et al. 2013 |
| 60 | *C. wangi* sp. nov. | *C. w. wangi* ssp. nov. | Nada2, Hainan, China (ND2) | CIB091453~CIB091468 | KC875805~KC875820 | 16 | 19.51 | 109.48 | Huang et al. 2013 |
| 61 | *C. wangi* sp. nov. | *C. w. wangi* ssp. nov. | Macao, China (MC) | / | / | / | 22.158855 | 113.577999 | This study |
| 62 | *C. wangi* sp. nov. | *C. w. wangi* ssp. nov. | Yizhang, Hunan, China (YZ) | / | / | / | 24.942264 | 112.93057 | Deng and Ye 1997 |
| 63 | *C. wangi* sp. nov. | *C. w. wangi* ssp. nov. | Funing, Yunnan, China (FN) | / | / | / | 23.484035 | 105.793296 | Yang and Rao 2008 |
| 64 | *C. irawadi* |  | Mt. Gaoligong, Yunnan, China (GLG) | HC201006290~HC201006291 | / | 2 | 26.42 | 98.9 | Huang et al. 2013 |
| 65 | *C. irawadi* |  | Xishuangbanna, Yunnan, China (XSBN) | HC201006292 | / | 1 | 22.01 | 100.8 | Huang et al. 2013 |
| 66 | *C. irawadi* |  | Nabang, Yunnan, China (NB) | KIZ 059191, NB20180905 | / |  | 24.7631 | 97.5708 | Liu et al. 2021 |
| 67 | *C. irawadi* |  | Taiping, Yunnan, China (TP) | KIZ HBH20200913, HBH20200914 | / |  | 24.4222 | 97.5511 | Liu et al. 2021 |
| 68 | *C. irawadi* |  | Longchuang, Yunnan, China (LC) | / | / |  | 24.190523 | 97.748512 | Yang and Rao 2008 |
| 69 | *C. irawadi* |  | Lushui, Yunnan, China (LS) | / | / |  | 25.805479 | 98.936521 | Yang and Rao 2008 |
| 70 | *C. irawadi* |  | Baoshan, Yunnan, China (BS) | / | / |  | 25.108434 | 99.130071 | Yang and Rao 2008 |
| 71 | *C. irawadi* |  | Menglian, Yunnan, China (ML) | / | / |  | 22.354561 | 99.524849 | Yang and Rao 2008 |
| 72 | *C. irawadi* |  | Yangbi, Yunnan, China (YB) | / | / |  | 25.670565 | 99.951768 | Yang and Rao 2008 |
| 73 | *C. irawadi* |  | Sagaing, Myanmar (SG) | / | / |  | 23.5743 | 95.7377 | Zug et al. 2006 |
| 74 | *C.* cf. *versicolor* |  | Bago1, Myanmar (BG1) | CAS 206551 | DQ289470 |  | 17.063 | 96.2519 | Zug et al. 2006 |
| 75 | *C.* cf. *versicolor* |  | Bago2, Myanmar (BG2) | USNM-GZ 35987 | DQ289471 |  | 17.5833 | 96.5667 | Zug et al. 2006 |
| 76 | *C.* cf. *versicolor* |  | Ayeyarwadi, Myanmar (AY) | CAS 205008 | DQ289469 |  | 16.2801 | 94.7706 | Zug et al. 2006 |
| 77 | *C.* cf. *versicolor* |  | Mawlamyine, Myanmar (ML) | CAS 222606 | DQ289472 |  | 16.3433 | 97.6874 | Zug et al. 2006 |
| 78 | *C.* cf. *versicolor* |  | Kyaik, Myanmar (KY) | USNM-GZ 35783, USNM-GZ 35815, USNM-GZ 35831 | DQ289475, DQ289474, DQ289473 |  | 17.320551 | 97.007713 | Zug et al. 2006 |
| 79 | *C.*cf. *versicolor* |  | Rakhine, Myanmar (RK) | CAS 204991 | DQ289476 |  | 17.7177 | 94.5321 | Zug et al. 2006 |
| 80 | *C.* cf. *versicolor* |  | Shan, Myanmar (SS) | CAS 230481 | DQ289477 |  | 21.1077 | 96.3577 | Zug et al. 2006 |
| 81 | *C.* cf. *versicolor* |  | Yangon, Myanmar (YG) | CAS 208157 | DQ289478 |  | 16.85955 | 96.13864 | Zug et al. 2006 |
| 82 | *C.* cf. *versicolor* |  | Chiang Khong, Thailand (CK) | / | / |  | 20.2498 | 100.4106 | Tantrawatpan et al. 2021 |
| 83 | *C.*cf. *versicolor* |  | Kenthao, Lao PDR(KT) | / | / |  | 17.8336 | 101.5555 | Tantrawatpan et al. 2021 |
| 84 | *C.* cf. *versicolor* |  | Tha Li, Thailand (TL) | / | / |  | 17.8153 | 101.5538 | Tantrawatpan et al. 2021 |
| 85 | *C.* cf. *versicolor* |  | Tha Bo, Thailand (TB) | / | / |  | 17.893 | 102.6011 | Tantrawatpan et al. 2021 |
| 86 | *C.* cf. *versicolor* |  | Mueang Bueng Kan, Thailand (MB) | / | / |  | 18.379 | 103.6347 | Tantrawatpan et al. 2021 |
| 87 | *C.* cf. *versicolor* |  | Mueang Nakhon, Thailand (MK) | / | / |  | 17.4448 | 104.7461 | Tantrawatpan et al. 2021 |
| 88 | *C.* cf. *versicolor* |  | Mueang Mukdahan, Thailand (MM) | / | / |  | 16.5144 | 104.706 | Tantrawatpan et al. 2021 |
| 89 | *C.* cf. *versicolor* |  | Chanuman, Thailand (CM) | / | / |  | 16.2342 | 104.9991 | Tantrawatpan et al. 2021 |
| 90 | *C.*cf. *versicolor* |  | Khemmarat, Thailand (KH) | / | / |  | 16.0861 | 105.0686 | Tantrawatpan et al. 2021 |
| 91 | *C.*cf. *versicolor* |  | Khong Chiam, Thailand (KC) | / | / |  | 15.3189 | 105.4956 | Tantrawatpan et al. 2021 |
| 92 | *C.* cf. *versicolor* |  | Huay Xai, Lao PDR(HX) | / | / |  | 20.2631 | 100.4336 | Tantrawatpan et al. 2021 |
| 93 | *C.* cf. *versicolor* |  | Nakhon Luang Prabang, Lao PDR(NLP) | / | / |  | 19.8703 | 102.1147 | Tantrawatpan et al. 2021 |
| 94 | *C.* cf. *versicolor* |  | Sanakham, Lao PDR(SK) | / | / |  | 18.0334 | 102.6255 | Tantrawatpan et al. 2021 |
| 95 | *C.* cf. *versicolor* |  | Hadxayfong, Lao PDR(HF) | / | / |  | 17.8983 | 102.6217 | Tantrawatpan et al. 2021 |
| 96 | *C.* cf. *versicolor* |  | Pakxon, Lao PDR(PK) | / | / |  | 18.3964 | 103.6558 | Tantrawatpan et al. 2021 |
| 97 | *C.* cf. *versicolor* |  | Thakhek, Lao PDR(TH) | / | / |  | 17.4553 | 104.7871 | Tantrawatpan et al. 2021 |
| 98 | *C.* cf. *versicolor* |  | Kaysone Phomvihane, Lao PDR(KP) | / | / |  | 16.6055 | 104.7715 | Tantrawatpan et al. 2021 |
| 99 | *C.* cf. *versicolor* |  | Pakse, Lao PDR(PK) | / | / |  | 15.1008 | 105.8523 | Tantrawatpan et al. 2021 |
| 100 | *C. versicolor* |  | Kalapet,India | CESL036, CESL1086, NCBS AT102 | MZ489207-MZ489209 |  | 12.02909 | 79.8503 | Gowande et al. 2021 |

**Table S2.** Comparison of measurement data between different populations of *Calotes wangi* sp. nov. and *C.* cf. *versicolor* populations. Morphometric measurements are in mm. For measurement methods and abbreviations, see the Materials and methods.

| Samples | | *Calotes* cf. *versicolor* | | *C.* cf. *versicolor* | | *C.* cf. *versicolo* | | *C.* cf. *versicolo* | | *C.* cf*. versicolo* | | *C.* cf. *versicolo* | | *C. irawadi* | | *C. irawadi* | | | *C.wangi* sp. nov. | | *C.wangi* sp. nov. | | *C. wangi wangi* ssp. nov. | | *C. wangi wangi* ssp. nov. | *C. w. hainanensis* ssp. nov. | | *C. w. hainanensis* ssp. nov. |
| --- | --- | --- | --- | --- | --- | --- | --- | --- | --- | --- | --- | --- | --- | --- | --- | --- | --- | --- | --- | --- | --- | --- | --- | --- | --- | --- | --- | --- |
| Population | Nat-Ma-Taung | | Nat-Ma-Taung | | Moyingyi | | Moyingyi | | Thai-east | | Thai-east | | Myanmar and SW. China | | Myanmar and China | | China and Vietnam | China and Vietnam | | mainland | | mainland | | central and southern Wuzhi Mountains on Hainan Island | | | central and southern Wuzhi Mountains on Hainan Island | |
| References | | Zug et al. (2006) | | Zug et al. (2006) | | Zug et al. (2006) | | Zug et al. (2006) | | Zug et al. (2006) | | Zug et al. (2006) | | Zug et al. (2006), Liu et al. (2021) and this study | | Zug et al. (2006), Liu et al. (2021) and this study | | | This study | | This study | | This study | | This study | This study | | This study |
| Sex | | ♀5 | | ♂3 | | ♀3 | | ♂7 | | ♀2 | | ♂11 | | ♀7 | | ♂9 | | | ♀92 | | ♂95 | | ♀78 | | ♂83 | ♀14 | | ♂12 |
| SVL | | 56.1~89.4 (79.4) | | 87.0~89.3 (88.3) | | 78.2~88.0 (83.4) | | 82.9~97.5 (91.4) | | 71.2~76.0 (73.6) | | 69.9~90.1 (81.2) | | 76~97(83.79) | | 75~98(84.92) | | | 66~105(84.3) | | 67~109(85.6) | | 66~105(84.3) | | 68~109(86.1) | 76~92(84.2) | | 67~95(82.1) |
| Tail | | / | | / | | / | | / | | / | | / | | 200~269(234.3) | | 217~271(244) | | | 151~329(239.3) | | 148~301(245.2) | | 179~268(236.0) | | 1482~301(242.2) | 151~329(257.8) | | 234~290(269.4) |
| HeadW | | 9.9~19.4 (17.1) | | 19.0~22.2 (21.0) | | 13.2~16.4 (14.4) | | 17.7~24.1 (20.1) | | 13.5~14.8 (14.2) | | 13.8~23.3 (18.9) | | 10.57~19.99(15.14) | | 13.08~20.12(15.65) | | | 11.46~18.81(14.12) | | 11.64~19.72(14.84) | | 11.46~18.81(14.07) | | 11.64~19.02(14.81) | 12.38~15.88(14.38) | | 12.2~19.72(15.10) |
| Interorb | | 6.4~9.3 (8.5) | | 9.6~10.3 (10.1) | | 8.1~9.9 (8.9) | | 9.1~10.0 (9.6) | | 9.1~9.3 (9.2) | | 8.9~11.6 (10.5) | | 8.7~13.64(11.23) | | 9.4~15.07(12.10) | | | 4.59~12.67(8.71) | | 6.08~12.89(8.90) | | 5.63~11.87(8.68) | | 6.76~12.89(8.96) | 4.59~12.67(8.89) | | 6.08~11.14(8.49) |
| HeadL | | 13.0~19.8 (18.1) | | 21.0~22.0 (21.7) | | 16.9~19.4 (17.9) | | 19.1~23.5 (21.0) | | 17.7~18.0 (17.9) | | 17.9~22.8 (20.0) | | 20.64~27.37(24.69) | | 20.14~29.41(24.77) | | | 14.1~26.41(21.96) | | 10.11~28.85(23.05) | | 14.1~26.41(21.83) | | 10.11~28.85(23.04) | 19.69~24.89(22.65) | | 19.06~26.98(23.11) |
| EyeEar | | 3.5~5.3 (4.8) | | 6.3~6.5 (6.4) | | 4.0~4.6 (4.2) | | 4.7~6.8 (6.1) | | 4.2~4.3 (4.3) | | 4.8~6.7 (5.8) | | 4.5~6.78(5.69) | | 4.61~7.31(5.79) | | | 4~7.41(5.40) | | 3.79~7.89(5.68) | | 4~7.41(5.38) | | 3.79~7.89(5.67) | 4.74~6.63(5.51) | | 4.12~7.5(5.79) |
| SnForeL | | 21.2~31.2 (28.2) | | 30.2~32.0 (31.2) | | 29.0~31.8 (30.7) | | 31.6~37.7 (35.4) | | 26.3~28.6 (27.5) | | 26.8~36.1 (32.5) | | 24.38~30.81(27.77) | | 25.82~33.49(29.04) | | | 2.84~7.78(4.66) | | 2.77~7.89(4.65) | | 21.44~36.27(27.87) | | 22.91~37.65(30.08) | 23.58~30.92(27.57) | | 22.47~32.89(28.40) |
| ForeLimbL | | / | | / | | / | | / | | / | | / | | 33.1~56.3(37.90) | | 35.45~44.99(40.02) | | | 31.11~49.12(40.29) | | 34.68~51.89(42.20) | | 31.11~49.12(40.35) | | 34.68~51.89(42.21) | 34.86~42.96(39.96) | | 36.71~46.42(42.16) |
| UparmL | | / | | / | | / | | / | | / | | / | | 13.79~17.55(15.69) | | 12.75~18.84(16.51) | | | 10.5~18.05(14.51) | | 11.39~19.51(14.78) | | 9.99~15.74(12.94) | | 9.81~16.33(13.41) | 11.88~14.99(13.19) | | 11.42~15.03(13.57) |
| LoArmL | | / | | / | | / | | / | | / | | / | | 11.9~14.81(13.35) | | 11.12~15.7(13.97) | | | 9.99~15.74(12.98) | | 9.81~16.33(13.43) | | 9.99~15.74(12.94) | | 9.81~16.33(13.41) | 11.88~14.99(13.19) | | 11.42~15.03(13.57) |
| HindLimbL | | / | | / | | / | | / | | / | | / | | 50.09~64.29(56.63) | | 54.31~64.49(60.43) | | | 53.8~69.77(61.70) | | 53.3~75.08(64.45) | | 53.8~69.77(61.59) | | 53.3~75.08(64.32) | 55.17~67.77(62.28) | | 58.17~71.9(65.41) |
| ForeLimbL/HindLimbL | | / | | / | | / | | / | | / | | / | | 0.58~0.98(0.67) | | 0.61~0.72(0.66) | | | 0.58~0.83(0.65) | | 0.6~0.73(0.65) | | 0.58~0.83(0.65) | | 0.6~0.71(0.66) | 0.61~0.68(0.64) | | 0.62~0.73(0.64) |
| UpLegL | | / | | / | | / | | / | | / | | / | | 17.3~22.47(19.63) | | 16.72~23.28(20.90) | | | 15.1~22.61(18.96) | | 9.82~24.98(19.63) | | 15.1~22.37(18.90) | | 9.82~23.65(19.46) | 17.27~22.61(19.36) | | 16.82~24.98(20.80) |
| CrusL | | / | | / | | / | | / | | / | | / | | 17.07~20.31(18.61) | | 16.82~21.52(19.02) | | | 14.23~20.28(17.67) | | 8.74~23.42(18.56) | | 14.23~20.28(17.60) | | 8.74~22.04(18.37) | 16.28~20.21(18.07) | | 17.03~23.42(19.85) |
| 4ToeLng | | / | | / | | / | | / | | / | | / | | 13.83~16.41(15.26) | | 13.27~17.2(15.83) | | | 7.06~19.54(16.91) | | 12.51~27.5(18.28) | | 7.06~19.54(16.90) | | 12.51~27.5(18.15) | 12.36~19.31(16.99) | | 16.77~24.04(19.16) |
| 4FingLng | | / | | / | | / | | / | | / | | / | | 8~12(9.5711) | | 7.95~11.5(10.07) | | | 8.9~18.45(11.83) | | 9.99~17.84(12.12) | | 8.9~14.01(11.77) | | 9.99~17.84(12.12) | 9.82~18.45(12.17) | | 10.36~16.99(12.16) |

**Supplementary figure S1.** Local enlarged phylogenetic tree structure corresponding to *Calotes wangi hainanensis* ssp. nov. (Lineage A) and *C. w. wangi* ssp. nov. (Lineage B), respectively. Numbers at the branch terminals and letters in parentheses correspond to the voucher number and abbreviation of localities in Table 1 and Table S1, respectively.


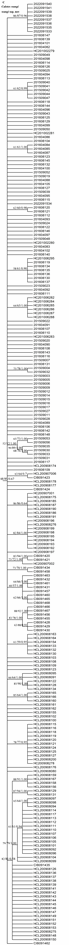


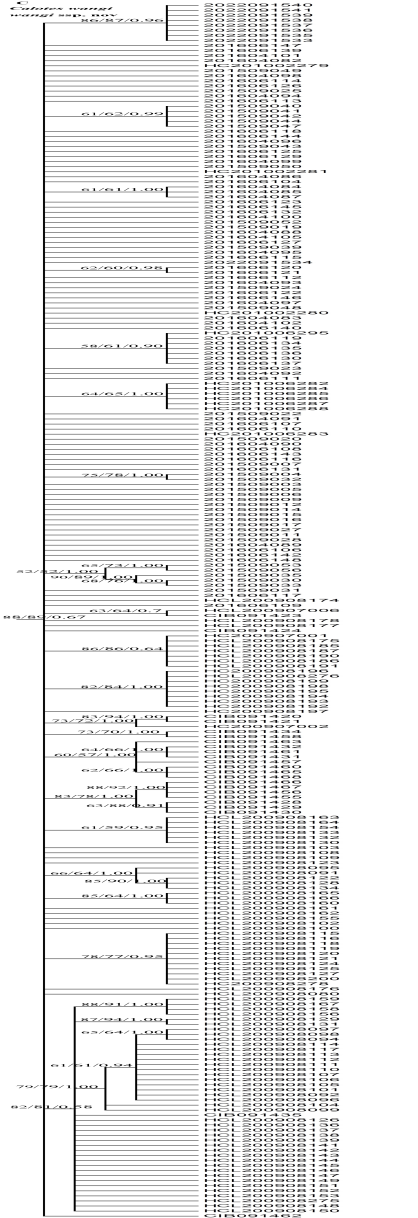

Supplement: Supplementary material 1 — Supplementary information [file zookeys-1187-063_article-110704__-s001.docx]
